# The Darker the Better: Identification of Chemotype Profile in Soroses of Local and Introduced Mulberry Varieties with Respect to the Colour Type

**DOI:** 10.3390/foods12213985

**Published:** 2023-10-31

**Authors:** Andreja Urbanek Krajnc, Jan Senekovič, Silvia Cappellozza, Maja Mikulic-Petkovsek

**Affiliations:** 1 Faculty of Agriculture and Life Sciences, University of Maribor, Pivola 10, 2311 Hoče, Slovenia; andreja.urbanek@um.si (A.U.K.); jan.senekovic1@um.si (J.S.); 2 Consiglio per la Ricerca in Agricoltura e l’Analisi dell’Economia Agraria, Centro di Ricerca Agricoltura e Ambiente (CREA-AA), Via Eulero 6a, 35143 Padua, Italy; silvia.cappellozza@crea.gov.it; 3 Biotechnical Faculty, University of Ljubljana, Jamnikarjeva 101, 1000 Ljubljana, Slovenia

**Keywords:** mulberry soroses, sugars, organic acids, phenolics, anthocyanins

## Abstract

Mulberries are the “essence of the past”, the so-called Proust effect, for the inhabitants of the sericultural regions who enthusiastically remember feeding silkworms with mulberry leaves and picking the different coloured fruits that were their favourite sweets in childhood. To determine the chemistry behind the colour and taste of mulberry soroses, the main metabolites of the local and introduced varieties were studied. The soroses were classified into five different colour types and the size parameters were determined. The main sugars identified were glucose and fructose, while the predominant organic acids were citric and malic acids, which were highest in the darker varieties, and fumaric and tartaric acids, which were highest in the lighter varieties. A total of 42 phenolic compounds were identified. The predominant phenolic acid was chlorogenic acid, followed by other caffeoylquinic acids and coumaroylquinic acids. The predominant anthocyanins were cyanidin-3-glucoside and cyanidin-3-rutinoside. According to PCA analysis, the colour types showed a clear chemotype character. The sweet taste of the yellowish-white soroses was defined by 49% fructose, followed by 45% glucose and 6% organic acids. The sour character of the black genotypes was characterised by a lower sugar and higher (11%) organic acid content. The colour- and species-dependent effect was observed in the proportion of caffeoylquinic acids and quercetin glycosides, which decreased with increasing colour intensity from 60% of the total to 7%, and from 17% to 1%, respectively. An upward trend was observed for flavanols (5% to 29%) and anthocyanins, which accounted for 62% of the total phenolics in black varieties. This article gives an insight into the metabolite composition of mulberry soroses as the sweets of choice between light and sweet and dark and sour.

## 1. Introduction

White mulberry (*Morus alba*, Moraceae) is native to south-western China, where it is widely cultivated, mainly for its leaf yield to feed the caterpillars of the silk-producing Lepidopteran insect *Bombyx mori* L. [1,2]. It was introduced into Europe from Sicily between the ninth and twelfth centuries [3,4,5,6,7,8,9]. The cultivation was later extended to other European countries and, over centuries, white mulberry became an integral part of the landscape, bearing witness to the sericultural past activity. 

Mulberry trees are unique in their sexual expression as they are both monoecious and dioecious, and their inflorescences are male or female catkins, appearing also in combinations of both male and female flowers. The small, multiple sweet infructescences (soroses) are a consolidation of drupelets with remnants of perianth [10]. The colour of the soroses is not a reliable distinguishing feature for identifying between black and white mulberry species, as the latter shows colours ranging from yellowish white to pink, dark red, and black, with different shading as they ripen [11]. The main components of mulberry soroses are primary metabolites, e.g., sugars, organic acids, and proteins, which reach values of 1.44 g/100 g [12,13,14], that are higher than in most berry fruits [15,16]. Numerous studies have demonstrated the presence of various bioactive components in mulberries, such as flavonoids, carotenoids, vitamins (A, B, E, K), polysaccharides, fatty acids, minerals, melatonin, and certain alkaloids [13,17]. Consumption of mulberry fruits has been associated with a lower risk of several chronic diseases such as cardiovascular and neurodegenerative diseases, certain cancers, diabetes type II, and osteoporosis [13,18,19]. 

Phenolic compounds play an important role in health due to their high antioxidant capacity, which combats oxidative stress in the human body [18,20]. Phenolic acids and flavonoids are the most abundant classes of phenolics in fruits and leaves of mulberry [21]. The predominant phenolic acid in white mulberry fruits is chlorogenic acid [22]. The major flavonoid compounds identified in mulberry fruit are rutin, quercetin rutinoside hexoside (morkotin A) and other quercetin derivatives, catechin, epicatechin, kaempferol hexoside, morin, naringenin derivatives, and anthocyanins in dark coloured genotypes [13,23,24]. 

Anthocyanins belong to the group of flavonoids and are responsible for the different colours of flowers and fruits, ranging from red and purple to blue [25]. They possess antioxidant, anti-inflammatory, and DNA-protective properties and are therefore associated with various health benefits [13,26]. The most abundant anthocyanins in white mulberry soroses are cyanidin-3-glucoside and cyanidin-3-rutinoside, followed by pelargonidin-3-glucoside, pelargonidin-3-rutinoside, cyanidin 3-*O*-sophoroside, petunidin-3-glucopyranoside, and peonidin 3-*O*-rutinoside in lower concentrations [13,27,28,29,30,31,32]. Qualitative and quantitative differences in the amounts of anthocyanins result in different colour types of the white mulberry [33]. Mulberries, especially the black and purple coloured ones, are a very rich source of anthocyanins, and white coloured mulberries are rich in phenolic acids [34]. The content of sugars and organic acids has an important influence on the taste characteristics of fruits and is also associated with a number of functions, such as sweetness, texture, and microbiological stability [35]. Large differences in sugar and organic acid content were found between the different varieties of *M. alba* by different authors [36,37,38]. In general, the glucose content of white mulberry was higher than the fructose content, while sucrose was found in low concentrations depending on the maturity stage [32,36,37,39]. In addition, white mulberry soroses have also been found to contain xylose, ketose, nystose, and inulin [37]. The most important organic acids in the fruits are malic and citric acid, followed by tartaric, succinic, and fumaric acids [36,40,41]. As mulberries are usually processed into fruit juices, syrup, molasses, or dried, it is important to pay attention to the composition of the phenolic compounds in relation to sugars and organic acids, as the processing into juice and molasses, as well as drying, negatively affect phenolics, leading to colour deterioration and browning [42,43]. With increasing citric acid content, mulberry syrup showed significantly higher antioxidant activity and dried fruits showed less colour deterioration [44,45,46].

Genotype is the most important factor in determining fruit chemical composition and antioxidant capacity [47]. The very long period of mulberry cultivation has led to the emergence of genotypes with different fruit characteristics through agronomic selection [48,49,50,51,52,53,54]. Morphological differences are strongly associated with different genotypes and the most important morphological traits include fruit size, shape, and colour [55,56]. Currently, there are few data on the nutraceutical characteristics of white mulberry fruits in Europe [57,58,59], apart from the recently published study by Truzzi et al. [32], which analysed different mulberry cultivars from the germplasm collection of CREA Padua, representing the pomological diversity in Italy in terms of fruit maturity. As relatively few studies deal with the characteristics of mulberries in relation to important metabolites, this leads to insufficient selection criteria. During our inventory of mulberry trees in different regions of Slovenia and Hungary, shoots of historical trees were taken for propagation in order to establish a mulberry collection and to investigate the genetic and biochemical characteristics of the existing mulberry gene pool. The screening of metabolites in the leaves allowed us to define superior genotypes of the local gene pool that have already proven their potential as forage [60,61,62]. 

The aim of this study was to determine the morphological and biochemical characteristics of different mulberry soroses obtained from the local Slovenian and Hungarian white mulberry varieties as well as from introduced sericultural and fruit varieties grown under the same continental conditions in relation to five different colour types, in order to define more precisely the chemistry behind the colour and taste of mulberry soroses.

## 2. Materials and Methods

### 2.1. Plant Material for Biochemical Analyses

Mulberry genotypes of five different colour types were chosen from the University of Maribor mulberry collection (φ: 46° 50′ 88.33″; γ: 15° 62′ 20.73″) which is organised into three sections: the first part is represented by sericultural *M. alba* varieties (‘Florio’, ‘Morettiana’, ‘Muki’) and the Japanese variety “Kokusou-20” obtained from the gene bank at the Consiglio per la Ricerca in Agricoltura e l’Analisi dell’Economia Agraria, Centro di Ricerca Agricoltura e Ambiente (CREA-AA), Padua, Italy. The second part comprises vegetatively propagated trees derived from the local historical Slovenian and Hungarian mulberries, which were obtained during the inventory of the mulberry gene pool. The third part of the collection is intended for growing recent varieties of *M. alba*, *M. nigra*, and *M. indica*, as well as hybrids of *M. alba* × *rubra* suitable for fruit production. 

**Figure 1:**
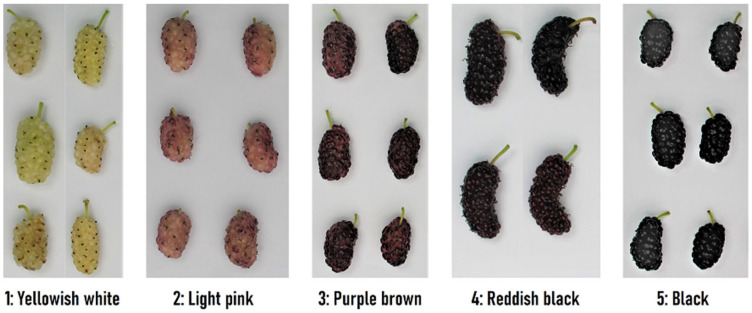
Five different colour categories of *Morus alba* soroses [63,64].

The mulberry trees for the experiments were selected from: (1) the original old Slovenian and Hungarian mulberry genotypes, and (2) species and introduced cultivars. The soroses werewith regard to their colour types categorized according to Urbanek Krajnc and Kozmos [63] and UPOV [64] descriptors into 5 colour categories: (1) yellowish white, (2) light pink, (3) purple brown, (4) reddish black, (5) black (Figure 1). The soroses (250 mL) from three to five trees of the same genotype were harvested twice between 15 June and 5 July 2021 at the time of full ripeness. The list of mulberry genotypes along with their location and geographic coordinates of historical mulberry trees are presented in Supplementary Table S1.

### 2.2. Measurements of the Size Parameters of the Mulberry Soroses 

In addition, the morphological characteristics (length, width, and peduncle length of the soroses) were analysed on 30 soroses of each genotype on two sampling dates in the laboratory using a stereomicroscope (Olympus SZ equipped with a camera and evaluated with Cell^A 2.4 software (Olympus BioSystems, Houston, TX, USA, 2006).

### 2.3. Determination of Individual Sugars and Organic Acids

Extraction, determination, and evaluation of sugars and organic acids were performed according to Sadar et al. [65]. Mixed fruit material (60 mg) was extracted in 4 mL double distilled water, incubated 30 min on an orbital shaker, and centrifuged for 15 min at 2800 rpm. Afterwards, 1.5 mL of extract was transferred to microtubes and centrifuged for 45 min at 14,000 rpm. Individual sugars, such as sucrose, glucose, and fructose, were analysed isocratically via HPLC using a Waters 2695 HPLC system and Refractometer Waters 410 (temperature 40 °C). The column was Phenomenex, Rezex RCM–Monosaccharide Ca^2+^ (300 × 7.8 mm). The column temperature was 65 °C. The mobile phase was double distilled water. Run time was 30 min, with flow rate of 0.4 mL/min. The organic acids in the samples extracted with water were analysed isocratically via HPLC using a Waters 2695 HPLC system and Waters 996 PDA detector (excitation: 210 nm). The column was Bio–Rad (Hercules, CA, USA), Aminex HPX–87H ion (300 × 7.8 mm). The solvent was 0.004 M H_2_SO_4_ and run time was 25 min at a flow rate of 0.6 mL/min.

### 2.4. Determination of Phenolics

Total phenolic compounds were determined spectrophotometrically, according to Urbanek Krajnc et al. [62]. Samples (2 ± 0.2 g) were extracted in 4 mL 95% methanol, incubated 30 min at room temperature in the dark and then centrifuged. From each sample, 100 μL of supernatant was collected and mixed with 200 μL of 10% Folin–Ciocalteu (FeC) reagent in microtubes. Precisely after 3 min, 800 μL of 700 mM Na_2_CO_3_ was added, vortexed and incubated 2 h at room temperature in the dark. The concentration of total phenolics was analysed spectrophotometrically, using a wavelength of 765 nm. Total phenolics were calculated as gallic acid equivalents using the regression equation between gallic acid standards (50 mM–2.5 mM gallic acid in 95% (vol/vol) methanol).

Furthermore, the methanolic extracts were subjected to a mass spectrometer (LTQ XL Linear Ion Trap Mass Spectrometer, Thermo Fisher Scientific, Waltham, MA, USA) with electrospray interface (ESI) operating in negative and positive ion mode. The procedures were previously described by Mikulic-Petkovsek et al. [66,67]. The analyses were carried out using full scan data-dependent MS2 scanning from *m*/*z* 110 to 1500. Column and chromatographic conditions were identical to those used for the HPLC–DAD analyses. The electrospray ionisation parameters were similar to those previously reported by Mikulic-Petkovsek et al. [67]. Spectral data were elaborated using the Xcalibur^TM^ 4.3 software (Thermo Scientific, Waltham, MA, USA).

The identification of compounds was confirmed by comparing their spectra, retention times, and fragmentation, as well as by adding the standard solution to the sample. Quantification was achieved by comparison with corresponding external standards (4-hydroxybenzoic acid, caffeic acid, *p*-coumaric acid, ferulic acid, quercetin rhamnosyl-hexoside, 3-caffeoylquinic acid, 4-caffeoylquinic acid, 5-caffeoylquinic acid, quercetin-3-rutinoside, quercetin-3-glucoside, quercetin rhamnosyl-hexoside, quercetin galactoside, kaempferol-3-glucoside, catechin, epicatechin, naringenin, procyanidin B1, cyanidin-3-glucoside, cyanidin-3-rutinoside, and pelargonidin-3-glucoside; all obtained from Sigma Aldrich, St. Louis, MO, USA) of known concentration. For the compounds for which the standards were not available, related compounds were used as standards. Therefore, protocatechuic acid was quantified in an equivalent of 4-hydroxybenzoic acid, caffeic acid hexoside in an equivalent of caffeic acid, *p*-coumaric acid hexoside in *p*-coumaric acid, 3-feruloylquinic acid and 5-feruloylquinic acid in ferulic acid, and 5-coumaroylquinic acid 1 and 2 and dicaffeoylquinic acid 1, 2, and 3 were quantified in an equivalent of 5-caffeoylquinic acid. In addition, quercetin dihexoside and quercetin malonylglucoside were quantified in an equivalent of quercetin-3-glucoside. Naringenin hexoside 1 and 2 were calculated in an equivalent of naringenin, and laricitrin hexoside in an equivalent of quercetin-3-glucoside. Kaempferol-3-rutinoside was further analysed in an equivalent of kaempferol-3-glucoside, and procyanidin dimer 1 and 2 in an equivalent of procyanidin B1. The contents of individual compounds were expressed in mg/100 g FW.

### 2.5. Statistics

The size parameters of mulberry infructescences were presented by mean and standard deviation (SD) and were statistically evaluated using one-way analysis of variance (ANOVA), followed by post hoc comparison according to Duncan. Letters describe significant differences among colour groups. The results of biochemical analyses of mulberry infructescences are shown as mean (average) values (± standard error, SE) of the analyses on two sampling dates according to their ripening stage between 15 June and 5 July 2021 by homogenising soroses of 250 mL, in which three to five trees were sampled and used as one sample. Measurements were performed at least four times for each sample and in duplicate. Assumptions of normality for all chemical traits were checked with Kolmogorov–Smirnov test. Furthermore, the one-way analysis of variance (ANOVA) was used to verify the differences between the effect of the colour type and chemical traits. The post hoc Duncan test was employed for chemical traits that were evaluated as significant (*p* < 0.05). Two-way analysis of variance (ANOVA) was conducted to identify the species dependency and the effect of different colour categories on the biochemical traits of mulberry soroses.

The Pearson correlation coefficient (Sig. 2-tailed) was calculated between evaluated chemical parameters with respect to the main anthocyanins (cyanidin-3-glucoside, cyanidin-3-rutinoside, pelargonidin-3-glucoside) to analyse the correlative relationship among the measured metabolites and to find out the most effective differentiating traits in respect to colour type. Principal component analysis (PCA) enabled us to conduct a comprehensive assessment of chemical traits by discriminating infructescences’ colour types with respect to reference varieties.

IBM SPSS Statistics 25 (New York, NY, USA; 2017), StatSoft, Inc. Statistica 8.0 (Victoria, Australia; 2007) and Past 3.17 software were used for statistical analysis [68].

## 3. Results

The main primary metabolites and phenolics were evaluated in soroses of selected local mulberry genotypes, and sericultural and fruit varieties, maintained in the mulberry collection, to determine how the various mulberry colour types differ in chemotype and which metabolites are the most important distinguishing markers of the colour category.

### 3.1. Infructescence Size Parameters

Observed morphological characteristics (stalk length, infructescence length and width) of different colour groups of mulberry soroses are presented in Table 1. The yellowish-white soroses were found to be predominantly globular in shape, while the black infructescences were more or less cylindrical. There were statistically significant differences in average stalk length among colour categories. Purple-brown infructescences had the longest stalk (8.6 mm), followed by black infructescences (8.2 mm). The lowest average stalk length was observed in light pink infructescences and yellowish-white soroses (Table 1). 

Significant differences were also observed in the length and width of the soroses in relation to their colour. The highest average length was measured in the black colour type (23.2 mm) and the lowest in yellowish-white ones (16.6 mm), similarly to what was reported by Ahmed et al. [69] who studied genotypic effects on morphological characterisation of fruit traits in relation to colour type. They observed maximum infructescence length and width in a black coloured genotype [69].

The average width of the soroses was 14.3 mm for the black category, while it was the smallest for the yellowish-white ones (12.0 mm) (Table 1). On average, the longest and the widest soroses were found for the black colour type, A large fruit size is almost always advantageous, as consumers have a marked preference for large fruits, and this also influences the price [70]. In addition, black mulberry fruits were described as attractive and juicy, with a good balance of sweetness and acidity, making them the best tasting fruits among mulberry varieties [69]. On the other hand, the average shortest stalk was observed in the yellowish-white infructescence types. Mulberries are commonly used for ice cream, spirits, sorbet, and bakery products, especially cakes. In this use, the stalk of the mulberry is removed because is fibrous and has an undesirable flavour [71]. Therefore, mulberries with white inflorescences would also be suitable for use in the food industry, as the stalks do not need to be removed. They are mainly used as dried fruit or for the production of fruit molasses, where stalks are undesirable [72]. 

### 3.2. Sugars and Organic Acids

The main sugars identified were fructose, glucose, and xylose, as they are also known to be predominant sugars in other berries [73]. When comparing different local and reference varieties, fructose was found in a range between 0.99 and 4.15 g/100 g FW. Glucose ranged from 0.88 g/100 g FW to 3.92 g/100 g FW. Both the highest and lowest values for glucose and fructose were found in the yellowish-white Hungarian genotypes SO 1035 and ZA 2044. Xylose was found to be up to 0.03 g/100 g FW (Table 2, Supplementary Table S2).

The contents of glucose and fructose were the highest in the light pink soroses and the lowest in the reddish-black and black colour types, with this trend being significant only for fructose. Similarly, Dimitrova et al. [37] found that fructose and glucose were the predominant sugars in white mulberry fruits. Fructose content was slightly higher than the glucose content, which is in agreement with our study and the study by Sánchez et al. [74].

A similar trend was observed for xylose, which was significantly lowest in the black genotypes and highest in the light pink genotypes (Table 2, Supplementary Table S2), which is in agreement with the study of Chen et al. [75]. In the present study, sucrose was below the detection limit, which is in contrast to the results of Gungor and Sengul [76], who detected sucrose, albeit at low contents ranging from 1.57 to 4.36 mg/100 g FW. The reason why sucrose was not determined in the present study could be the maturity stage of the fruits, as the contents of sucrose in mulberry decreases as maturity progresses [56].

We were able to identify seven organic acids. The predominant ones were citric acid (up to 1.06 g/100 g FW in *M. nigra*), succinic acid (up to 0.41 g/100 g FW in ZA 2047), and malic acid (0.03–0.18 g/100 g FW), followed by lactic, fumaric, tartaric, and acetic acids (Table 2, Supplementary Table S2).

The reddish-black and black infructescences had significantly higher citric acid contents, while the yellowish-white soroses had the lowest contents. Eyduran et al. [47] found higher contents of citric acid in *M. nigra* compared to *M. alba*, which reached values of 1.03 g/100 g. This is consistent with our study, as the average citric acid content in *M. nigra* infructescences was 1.06 mg/100 g FW. In *M. alba*, citric acid content analysed by Eyduran et al. [47] was lower and reached values of 0.7 mg/100 g, which is also in accordance with the results of the present study.

There were no significant differences in malic acid among the colour types. Succinic acid was the highest in the purple-brown genotypes and the lowest in the yellowish-white ones. Lactic acid, similar to citric acid, was significantly the highest in the reddish black and black genotypes. The opposite trend was observed for fumaric acid, which was significantly the highest in the lighter genotypes, and lowest in the reddish-black and black varieties. Tartaric acid was the highest in the light pink colour category. 

Acidity, which plays an important role in the perception of fruit quality, affects not only the sour taste of the fruit, but also sweetness, by masking the taste of sugars [77]. The proportions of individual acids are also important. Citric acid masked the perception of sucrose and fructose [78,79], while malic acid enhanced sucrose perception. Furthermore, the different taste of the yellowish-white and the light pink soroses is probably influenced by the higher content of fumaric acid and tartaric acid. Tartaric acid and fumaric acid are more astringent than citric acid or malic acid [80]. The above results reflect that colour types have their specific composition of sugars and organic acids, which determine their taste. The sugar organic acid ratio was highest in yellowish-white (15.48) and lowest in black genotypes (8.03) (Table 2, Supplementary Table S2).

Generally, black and red mulberry infructescences have lower total sugar content compared to white mulberries, which are much sweeter [81]. In this study, this is evidenced by the higher value of the sugar:organic acid ratio in the yellowish-white and light pink infructescences compared to the black and reddish-black infructescences. Similar results were reported for black elderberries, which had a lower sugar content compared with the uncoloured elderberry variety *Sambucus nigra* var. *viridis* [82]. 

When comparing different species, the content of citric and acetic acids was particularly high in *M. nigra*, reaching values of 1058.76 and 132.44 mg/100 g, respectively, similar to those reported by Jiang et al. [83] The hybrids of *M. alba* × *M. rubra* were in the middle range for citric and acetic acid contents, but had high levels of malic and lactic acid, reaching values of 135.40 mg/100 g and 175.16 mg/100 g in the French hybrid compared to *M. alba* and *M. nigra*, respectively. *Morus indica* genotypes also showed medium character. The above results are supported by two-way analysis of variance (Supplementary Table S3). The contents of xylose, citric acid, and fumaric acid were significantly influenced by the colour. Glucose, fructose, citric, and tartaric acids were dependent on the species. The combined effect of species and colour was reflected in significantly different levels of citric acid. 

The results discussed above clearly show that the sugar and organic acid compositions are dependent on both species and colour. The nature and concentration of organic acids are important factors influencing the organoleptic properties and can maintain the nutritive value of fruit.

### 3.3. Phenolics

A total of 42 phenolic compounds were identified, and qualitative and quantitative differences between different colour types were studied. The identified phenolic compounds are listed together with the assigned molecular ions and their fragmentation pattern in Supplementary Table S4. Total phenolics ranged from 205.24 g/100 g FW in the local variety VE 2620 to 536.38 g/100 g FW in the variety *M. indica* “Shin Tso”. There was a gradual significant trend of increase in total phenolic content from light coloured infructescences, with an average content of 231.06 g/100 g FW in yellowish-white genotypes, to darker infructescences with an average content of 435.25 g/100 g in black varieties (Table 3, Supplementary Table S5). These results are within the range of previous studies. The average amount of total phenolics content determined in the study by Farahani et al. [14] was 430.47 mg/100 g FW. The highest average total phenolics content in our study was determined in black infructescences and the lowest total phenolics content was determined in yellowish-white infructescences. Other authors also reported that total phenolics content levels obtained from the black coloured mulberries were several times higher than the content of phenolics found in the light pink and yellowish-white mulberries [17,32].

The predominant phenolic acids were caffeoylquinic acids, followed by *p*-coumaroylquinic acids, caffeic acid, *p*-coumaric acid derivatives, and feruloylquinic acids. The predominant caffeoylquinic acid derivative was chlorogenic acid (5-caffeoylquinic acid), followed by 3-caffeoylquinic acid, 4-caffeoylquinic acid, and dicaffeoylquinic acid. Chlorogenic acid is the predominant phenolic acid in all fruit colour types, which is consistent with other authors reporting values in the range of 5.3–17.3 mg/100 g DW [13,84,85]. In our study, chlorogenic acid ranged from approximately 7 to 15 mg/100 g FW depending on the colour type, and were significantly the highest in reddish-black infructescences and the lowest in yellowish-white and light pink soroses (Table 3). Due to the high content of the chlorogenic acid, mulberry fruits may have many positive health benefits. The antioxidant properties of chlorogenic acid have been demonstrated in numerous studies, including the ability to induce cardioprotective effects, anti-tumour activity, and even neuroprotective effects [86].

3-Caffeoylquinic acid ranged from 0.2 to 11.7 mg/100 g FW and was significantly the highest in black infructescences and the lowest in yellowish-white ones (Table 3). Similarly, dicaffeoylquinic acid derivatives showed an upwards trend in content from the yellowish-white to the black colour types. Coumaroylquinic and feruloylquinic acids were the lowest in yellowish-white and significantly the highest in reddish-black and/or black colour types (Table 3). 

Caffeic acid did not differ significantly among the colour types, whereas caffeic acid derivatives showed an upwards trend in being the lowest in yellowish-white and the highest in reddish-black infructescences. Among the *p*-coumaric acid derivatives, the *p*-coumaric acid hexoside was significantly the highest in reddish-black and the lowest in purple-brown varieties (Table 3). The highest average content of all identified groups of phenolic acids was observed in the black or reddish-black infructescences. Our results are partially in agreement with other articles analysing the metabolite content of mulberry soroses [13,17,23,32,36,84]. Truzzi et al. [32] analysed 4-hydroxybenzaldehyde, protocatechuic acid glucoside, caffeoylquinic acid isomers (caffeoylquinic acid), dicaffeoylquinic acid (dicaffeoylquinic acid), caffeoyl glucose isomers, and caffeoyl and dicaffeoyl methylquinate isomers. Among the hydroxycinnamic acids, sinapinic acid and coumaroylquinic acid were identified. Yuan and Zhao [13] reviewed the following components of white mulberry infructescences: chlorogenic acid, ferulic acid, coumaric acids, cinnamic acid, gallic acid, *p*-hydroxybenzoic acid, syringic acid, protocatehuic acid, and vanillic acid. Of the phenolic acids of the reviewed studies, we did not determine 4-hydroxybenzaldehyde, *p*-hydroxybenzoic acid, cinnamic acid, gallic acid, syringic acid, or vanillic acid. Similar to the study by Natić et al. [17], we determined protocatechuic acid, chlorogenic acid, caffeic acid, *p*-coumaric acid, and ferulic acid or their derivatives. In contrast to the Natić study, we did not determine gallic acid, *p*-hydroxybenzoic acid, ellagic acid, or gentisic acid [17]. 

The most abundant flavonoids in mulberry fruits are anthocyanins, followed by flavonols (quercetin and kaempferol glycosides), flavanols (catechin, epicatechin and procyanidin s), flavanons, which are represented by naringenin derivatives, laricitrin hexoside, and quercetin rhamnosyl-hexoside. Quercetin derivatives were the most diverse phenolic group, showing a significant upwards trend in content from yellowish-white to black, besides quercetin malonylglucoside, which showed the opposite trend and was significantly the lowest in the black colour type. The predominant was quercetin-3-rutinoside, followed by quercetin malonylglucoside, quercetin-3-glucoside, quercetin-rutinoside hexoside (morkotin A), quercetin rhamnosyl-hexoside and quercetin dihexoside, quercetin-3-galactoside, quercetin-3-xyloside, and quercetin (Table 4, Supplementary Table S6). Quercetin-3-rutinoside (rutin) ranged from 1.26 to 2.58 mg/100 g FW from yellowish-white to black soroses (Table 4), respectively, which is in agreement with the results of previous studies, which determined rutin in a range between 0.06 and 7.73 mg/100 g FW [13,17,87]. The second most predominant representative of quercetin and its derivatives was quercetin malonylglucoside, in a range between 0.65 to 1.29 mg/100 g FW (Table 4). We previously determined quercetin malonylglucoside to be the most abundant flavonol in *M. alba* leaves [61]. Quercetin malonylglucoside was also determined in high proportions by Truzzi et al. [32]. Among the quercetin derivatives, it is the only representative whose content is significantly lower in the black coloured varieties than in the yellowish-white varieties. Xing et al. [88] reported that the petals of yellow variety of Asian cotton (*Gossypium arboreum* L.) have significantly higher quercetin malonylglucoside content compared to purple varieties, which are high in anthocyanins. Furthermore, Assefa et al. [89] reported that leaf samples of the analysed lettuce cultivars differed from other cultivars during bolting as they had a significantly higher content of quercetin malonylglucoside compared to other cultivars. These observations suggest that the colour may be influenced in part by the different quercetin malonylglucoside profile. It is likely that it also has an influence on mulberry fruit colour. Further studies would be needed to confirm this hypothesis.

Further predominant quercetin glucosides were quercetin-3-glucoside (0.38 to 0.45 mg/100 g FW) and quercetin-rutinoside hexoside (morkotin A, 0.09–0.20 mg/100 g FW). Jin et al. [90] reported quercetin-3-glucoside contents of 1.0 mg/100 mg. Morkotin A was reported by Ju et al. [91] in a range between 0.6 to 2.1 mg/100 g DW, which coincides with our results when taking into account the average water content of 45.6% measured in our study. Both quercetin rhamnosyl-hexoside and quercetin dihexoside reached contents up to 0.13 mg/100 g FW in reddish-black varieties. Quercetin-3-galactoside, quercetin-3-xyloside, and quercetin were found in contents below 0.08 mg/100 g FW (Table 4).

Kaempferol derivatives were represented by kaempferol-3-rutinoside, ranging from 0.05 mg/100 g FW in yellowish-white to 0.08 mg/100 g FW in black varieties, and kaempferol hexosides ranging from 0.02 mg/100 g FW in purple-brown to 0.08 mg/100 g FW in light pink varieties (Table 4, Supplementary Table S6). Considering that the average water content of the soroses was 45.6%, the kaempferol contents of the present study are in agreement with Butkhup et al. [23], who determined kaempferol derivatives in the range of 0.24 to 1.61 mg/100 g DW. However, several authors reported higher values compared to our results. Kaempferol glucoside was determined at a concentration of 1.623 mg/100 g FW by Jin et al. [90]. Sánchez-Salcedo et al. [12] reported kaempferol 3-rutinoside in a range between 2 and 14 mg/100 g DW. In addition, Ju et al. [91] determined kaempferol-3-rutinoside content of 5.3 mg/100 g DW, kaempferol 3-glucoside content of 3.1 mg/100 g DW, and kaempferol-malonyl-glucoside content of 4.1 mg/100 g DW. Flavanols represented by procyanidin dimers, catechin, and epicatechin were significantly the highest in black varieties and the lowest in yellowish-white varieties. We found procyanidins ranging from 1.11 mg/100 g FW to 116 mg/100 g FW in black infructescence types (Table 4). Truzzi et al. [32] found higher contents of up to 161 mg/100 g FW. In the flavonoid biosynthetic pathway, proanthocyanidins are the precursors of a branch of the anthocyanidin biosynthetic pathway. Proanthocyanidins and anthocyanins are derived from the same precursor, anthocyanidin, and share a common biosynthetic process for the conversion of phenylalanine to anthocyanidin [92]. Thus, we can conclude that the flavonoid metabolic pathway is more active in the darker infructescences. We also found that procyaninidin dimer 1 was higher in the yellowish-white infructescences than in light pink and purple-brown infructescences. This reflects a bottleneck in the conversion of procyanidins to anthocyanins, which has been explained in other berry fruits by the low expression of anthocyanidin synthase genes [93].

Three naringenin hexosides were determined among the flavanones, which ranged from 0.0001 to 0.06 mg/100 g FW. In yellowish-white varieties, naringenin hexoside 1 was the lowest, whereas naringenin hexosides 2 and 3 were the highest (Table 4). Chon et al. [94] determined naringenin at an average level of 0.093 mg/100 g, which is slightly higher than in our study. In the present study, the black infructescences had the highest total content of naringenin derivatives, reaching values of 0.075 mg/100 g FW. Moreover, black infructescences had the highest contents of total phenolics and anthocyanins. This high correlation is due to the fact that naringenin is a general precursor of flavonols, anthocyanins, proanthocyanidins, flavones, and isoflavones [95]. 

The diversity of colours of white mulberry soroses is an interplay of qualitative and quantitative differences of anthocyanins [32,33], which we found below the detection level in yellowish-white infructescences, and increased gradually by more than 100 times from light pink (2.86 mg/100 g FW), purple-brown (10.13 mg/100 g FW), reddish-black (140.01 mg/100 g FW), to black coloured genotypes (250.95 mg/100 g FW), which is in agreement with other authors [32,38,96].

The predominant anthocyanin was cyanidin-3-glucoside in a range from 2.34 to 165.77 mg/100 g FW, followed by cyanidin-3-rutinoside ranging from 0.24 to 69.90 mg/100 g FW. Pelargonidin-3-glucoside was determined in contents between 0.28 and 15.28 mg/100 g FW (Table 4). Cyanidin-3-sophoroside, pelargonidin-3-rutinoside, peonidin-3-rutinoside, and petunidin-3-glucoside were found in traces only in reddish-black and black varieties, and were not statistically evaluated (Table 4, Supplementary Table S6).

The results are in agreement with articles of Yuan and Zhao [13] and Truzzi et al. [32], which reported that cyanidin-3-*O*-glucoside was the most abundant, followed by cyanidin-3-*O*-rutinoside and pelargonidin-3-*O*-rutinoside. In contrast, Chen et al. [38] did not find cyanidin-3-*O*-rutinoside in any of the Chinese cultivars included in the experiment. Other authors also reported pelargonidin-3-glucoside in lower contents in white mulberry soroses [13,27,28]. In addition, Truzzi et al. [32] also found cyanidin 3-*O*-sophoroside and peonidin 3-*O*-rutinoside, which in our samples determined in traces. Du et al. [96] also identified cyanidin-3-*O*-rhamnopyranosyl-glucopyranoside and cyanidin-3-galactopyranoside, while Sheng et al. [29] detected petunidin-3-glucopyranoside in mulberry fruit. 

Among the reference varieties, ‘Morettiana’ and ‘Florio’ had the lowest contents of all compounds, which is in agreement with Truzzi et al. [32]. The extremely high variation in the anthocyanidin content has already been reported in other studies [13,29,32,38,58,96,97,98]. In the present study it is certainly due to the different genotypes, since the 56 genotypes of local origin, the sericultural reference, and fruit varieties included in the study were grown under the same pedoclimatic conditions of the germplasm collection of the University of Maribor. When comparing the contents of certain phenolics in mulberry fruit with other studies, we have to consider that fruit ripening is a complex process that can vary according to geographical origin and plant genetics.

In our study, cyanidin-3-glucoside reached 165.77 mg/100 g FW in black varieties. Truzzi et al. [32] found the contents ranging from 0.3 mg/100 g FW in ‘Morettiana’ to 205.8 mg/100 g FW in ‘Queensland Black’ variety. Cyanidin-3-rutinoside ranged from 0.47 mg/100 g in ‘Morettiana’to 156 mg/100 g FW in “Queensland Black”. We found cyanidin-3-rutinoside in contents of up to 182.85 mg/100 g FW in *M. alba* “Big Ten”. Truzzi et al. [32] reported pelargonidin-3-rutinoside of up to 0.72 mg/100 g FW, while in our study pelargonidin-3-rutinoside reached levels of 15.76 mg/100 g FW. The low values of anthocyanins in yellowish-white infructescences reflect the low expression of key genes of anthocyanidin synthesis and, consequently, low enzyme activity, which has been previously reported for berry fruits. For example, Zorenc et al. [93] could not detect anthocyanins in the white currant cultivar ‘Zitavia’, which had very low CHS/CHI activity and ANS expression, while FHT and DFR activities could not be detected. Similarly, Zorenc et al. [99] reported significantly lower expression levels of key structural genes in albino bilberry skins compared to the common blue type. Among them, the most affected genes were FGT, FHT, ANS, and CHS, followed by CHI, DFR, LAR, and ANR, while F3′5′H gene expression was slightly higher in albino *Vaccinium myrtillus* [99]. Moreover, the F3H expression level in white mulberry was shown to have a positive and close relationship with anthocyanin content during the anthocyanin-rich fruit ripening process, while it had a negative correlation with anthocyanin content in the *M. alba* genotype LvShenZi, whose fruits are white and do not undergo anthocyanin accumulation during fruit ripening [95]. From the above articles it can be concluded that the low content of anthocyanins in lighter soroses of the white mulberry is influenced by gene expression and enzyme activity. Further research would be needed to explain this phenomenon in more detail. 

Two-way analysis of variance showed that the contents of most compounds were significantly influenced by the colour. The contents of caffeic acid derivatives, caffeoylquinic acids, *p*-coumaric acid derivatives, coumaroylquinic acid derivatives, feruloylquinic acids, quercetin-3-rutinoside, quercetin dihexoside, kaempferol hexoside, laricitrin hexoside, isorharmnetin hexoside, and anthocyanins were dependent on the species. The combined effect of species and colour was reflected in significantly different levels of caffeic and *p*-coumaric acid derivatives, chlorogenic acid, quercetin-3-galactoside, quercetin-3-xyloside, morkotin A, quercetin dihexoside, kaempferol hexoside, keampferol-3-rutinoside, and cyanidin-3-glucoside (Supplementary Table S3).

### 3.4. Correlations between Anthocyanins and Other Metabolites in Terms of Colour Type

To determine the changes in metabolites in relation to colour type, Pearson’s correlation coefficient was calculated (Table 5). In general, a high positive correlation was found between anthocyanins and total phenolics (0.7 **). For phenolic acids, there was a strong correlation between anthocyanins and 3-caffeoylquinic acid (>0.7 **), 5-feruloylquinic acid (0.5 ** for cyanidin-3-glucoside and pelargonidin-3-glucoside), caffeic acid hexosides (>0.5 **), *p*-coumaric acid hexoside (0.5 ** for pelargonidin-3-glucoside), and *p*-coumaric acid (>0.45 ** for cyanidin-3-glucoside and cyanidin-3-rutinoside). A medium correlation was found between anthocyanins and chlorogenic acid and catechin (0.3 *). There was also a strong positive correlation between anthocyanins and quercetin (0.5 ** for cyanidin-3-glucoside and cyanidin-3-rutinoside), quercetin-rutinoside hexoside (morkotin A) (0.4 **), and quercetin-3-rutinoside (0.66–0.81 **). Similarly, Truzzi et al. [32] reported a strong correlation between the expression of quercetin derivatives and cyanidin 3-*O*-glucoside. The strong positive correlation between anthocyanins and epicatechin (0.4–0.5 **), as well as procyanidin dimer (0.6–0.9 **), reflects that these compounds are direct precursors of anthocyanins synthesis [92]. Furthermore, we were able to determine a strong correlation between anthocyanins and naringenin hexoside 1 (0.5 ** for cyanidin-3-glucoside and cyanidin-3-rutinoside), while the correlation with kaempferol-3-rutinoside was medium (0.3 *).

A tendency for sugar content to decrease with increasing anthocyanin contents was observed, which was confirmed by the negative Pearson correlation. A medium negative correlation was found between anthocyanins and xylose (−0.3 *). A strong negative correlation was found between anthocyanins and fumaric acid (−0.6 **), while there was a medium positive correlation with citric acid (0.4 * for pelargonidin-3-glucoside). A similar trend was also observed in the study by Zorenc et al. [93] using the white currant variety “Zitavia” as an example. Sugar not only provides carbon sources, skeletons, and glucosides for anthocyanin biosynthesis, but also increases the expression levels of biosynthetic structural genes and regulatory MYB genes [100]. Two reasons for the low sugar content and simultaneous high anthocyanin content in mulberry fruit are similar to those previously reported for Teinturier grapes. The first reason is the low activity of the enzymes responsible for sugar accumulation, namely sucrose-phosphate synthase and sucrose synthase. The second main reason is that anthocyanins are formed from anthocyanidins and sugar and the sugar was used to synthesise anthocyanins [82,101]. Kong et al. [102] described a complex relationship between sugar and anthocyanin accumulation. For example, carbon deficiency in both grapes and kiwifruit resulted in a greater decrease in anthocyanin content than sugar content, because carbon under carbon deficiency is preferentially used for sugar accumulation rather than anthocyanin biosynthesis. The anthocyanin pathway could serve as a carbon sink competing with sugar accumulation for carbon allocation at the organ and cell levels. Kong et al. [102] therefore concluded that the anthocyanin biosynthetic machinery in the skin of the Teinturier variant “Gamay Fréaux” probably competes for carbon at the expense of sugar accumulation. A further possible reason discussed in the article analysing green and black elderberry during the ripening process is that a decrease in sugar content during the last ripening stage may be due to the decline in their synthesis and remobilisation [82]. Further studies are needed for a more detailed description and interpretation of this phenomenon using the white mulberry as an example.

### 3.5. Chemotype Characterisation of Different Infructescence Colour Groups

**Figure 2:**
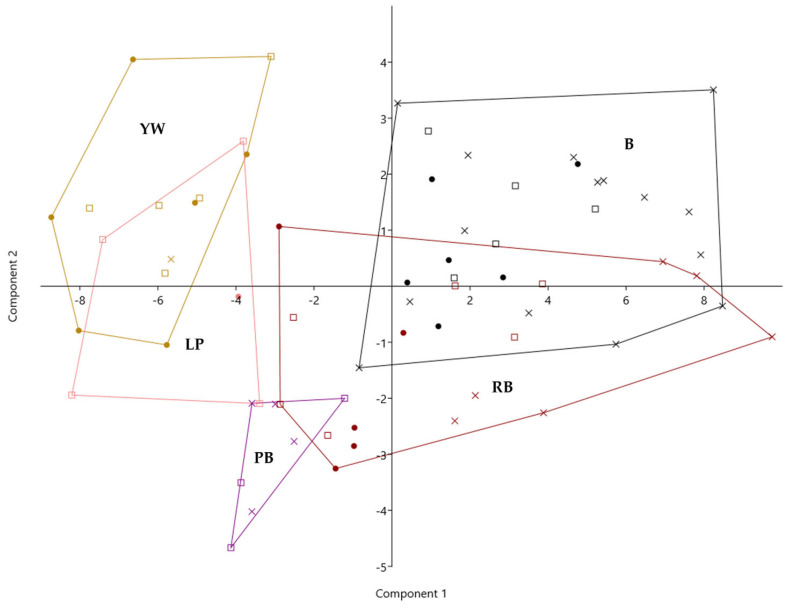
Principal component analysis of the chemical composition of five infructescence colour categories of mulberry trees from different Slovenian regions (squares) and Hungarian regions (dots) compared to traditional sericultural and fruit varieties (crosses). Legend: YW—yellowish white, LP—light pink, PB—purple brown, RB—reddish black, B—black colour type.

To enable a comprehensive assessment of the chemical composition of mulberry infructescences with respect to their colour, a principal component analysis (PCA) was conducted. The discriminant function 1, which accounts for 74.28% of the variance explained by the model, was weighed most strongly by citric and lactic acid, total phenolics, and anthocyanins. It was further negatively associated with sugars, fumaric acid, naringenin hexosides, and quercetin malonylglucoside (Figure 2, Supplementary Table S7). 

Thus, function 1 clearly separates yellowish-white varieties lacking anthocyanins and low in organic acids, total phenolics, and particular flavonoids, and high in caffeoylquinic acids, from darker fruit varieties, which are low in caffeoylquinic acids (<4%) and high in anthocyanins and flavanols. There was a 27% overlapping of yellowish-white with light pink varieties. The purple-brown colour type showed an intermediate chemotype. Reddish black showed the greatest variation and overlapped with both purple-brown and black varieties (Figure 2, Supplementary Table S7).

The second discriminant function accounts for another 13.35% of the variance and was positively associated with acetic acid, total phenolics, caffeic acid, epicatechin, naringenin hexosides, kaempferol hexoside, and anthocyanins. It was further negatively associated with tartaric acid, succinic acid, and fumaric acid, followed by 3-*p*-coumaroylquinic acid, caffeoylquinic and coumaroylquininc acid derivatives, and quercetin. There was a clear separation of purple-brown varieties, which were particularly high in succinic acid, from black varieties, which were particularly high in acetic acid and lactic acid, naringenin hexosides, quercetin, kaempferol derivatives, flavanols, and anthocyanins. The intermediate character of reddish-black varieties was most strongly weighted by *p*-coumaric acid hexoside, quercetin-dihexoside, caffeoylquinic and feruloylquinic acid derivatives, and citric acid (Figure 2, Supplementary Table S7).

**Figure 3:**
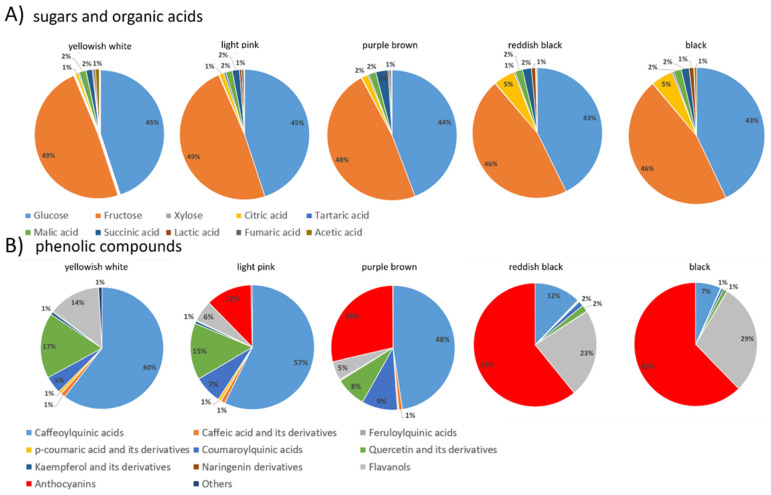
Chemotype characteristics of five different infructescence colour groups represented as pie charts. (**A**) Sugars and organic acids, (**B**) individual phenolics. For detailed contents of individual phenolics see Table 3.

In order to represent the colour chemotypes of the infructescences, the percentages of sugars, organic acids, and individual phenolics were determined for each infructescence colour type and are presented in the form of pie charts in Figure 3.

The yellowish-white soroses consisted of 49% fructose, followed by 45% glucose. Of the organic acids, malic and succinic acid represented 2%, and acetic and citric acid represented 1% each (Figure 3A). In addition, the yellowish-white fruits were characterised by the highest content of caffeoylquinic acids (60%), followed by quercetin derivatives (17%), flavanols (14%), and coumaroylquinic acids (5%) (Figure 3B).

Light pink soroses had the same ratio of fructose, glucose, malic, and succinic acids as the yellowish-white ones (Figure 3A). However, they were characterised by a lower content of caffeoylquinic acid derivatives (57%), quercetin glycosides (15%), and flavanols (6%) than the yellowish-white ones. They are also characterised by the presence of anthocyanins (12%) and a slightly higher content of coumaroylquinic acids (7%).

In the purple-brown soroses, fructose accounted for 48% and glucose for 44%, while succinic acid increased to 3% and malic and citric acids accounted for 2% of the total (Figure 3A). Among the individual phenolics, the purple-brown fruits had the highest proportion of caffeoylquinic acids (48%), followed by anthocyanins (29%), coumaroylquinic acids (9%), quercetin derivatives (8%), and flavanols (5%) (Figure 3B).

The reddish-black soroses showed a significant decrease in sugar content (46% fructose, 43% glucose) with a simultaneous increase in the proportion of organic acids. Citric acid was the predominant organic acid, accounting for 5% of the total, followed by succinic acid and malic acid, each accounting for 2% of the total sugars and organic acids (Figure 3A). Among the phenolics in the reddish-black genotypes, anthocyanins predominated (61%), followed by flavanols (23%) and caffeoylquinic acids (12%), while coumaroylquinic acids and quercetin derivatives accounted for 2% of the total phenolics (Figure 3B).

Black genotypes were similar to reddish black in the profile of sugars and organic acids (Figure 3A). There were obvious differences in the profile of phenolics. Black infructescences had the highest proportion of anthocyanins (62%), and flavanols (29%), while caffeoylquinic acids accounted for only 7% of the total phenolics (Figure 3B).

To our knowledge, this is the first article on primary and secondary metabolite screening of mulberry soroses, where multivariate analyses were performed in relation to five different colour types according to UPOV and Urbanek Krajnc and Kozmos [63,64]. This allowed us to clearly separate the colour groups and determine the major colour category discriminators, namely total phenolics, anthocyanins, citric acid, and lactic acid, which were higher in darker soroses, while fructose, xylose, and quercetin-malonylglucoside were significantly higher in lighter fruits.

Our future goal is to investigate the stability of the phenolic groups of the five different colour types after the drying process, as the drying technique and storage procedure can have negative effects, leading to colour deterioration and browning [42,43] (Deng et al. 2022, Li et al. 2022). The lighter genotypes and the purplish-brown varieties, in particular, show potential as a dried product.

## 4. Conclusions

The present study clearly shows that the different mulberry colour types differed in chemotype and that the most important distinguishing characteristics for the colour category were total phenols, anthocyanins, and citric and lactic acids, which were higher in darker soroses, while fructose, xylose, fumaric acid, and quercetin malonyl glucoside were significantly higher in lighter fruits.

Acidity, which plays an important role in the perception of fruit quality, influences not only the sour taste of the fruit, but also the sweetness, by masking the taste of the sugar. The proportions of the individual acids are also important. Citric acid masks the perception of sucrose and fructose, while malic acid enhances the perception of sucrose. In addition, the different taste of the yellowish-white and the light pink soroses is probably influenced by the higher contents of fumaric acid and tartaric acid, which are considered to be more astringent than citric or malic acid. When comparing the different species, the content of citric and acetic acid was particularly high in *M. nigra*. The hybrids of *M. alba* × *M. rubra* were in the middle range for the contents of citric and acetic acid, but had high contents of malic and lactic acid. These characteristics give black mulberry and *M. alba* × *M. rubra* hybrids a more concentrated aroma and flavour.

The combined effect of species and colour was reflected in significantly different levels of caffeic and *p*-coumaric acid derivatives, chlorogenic acid, morkotin A, quercetin di-hexoside, quercetin-3-galactoside, quercetin-3-xyloside, keampferol-3-rutinoside, kaempferol hexoside, and cyanide-3-glucoside. In the future, further research will be needed to explain the phenomenon of the low content of anthocyanins relative to their precursors in the lighter soroses of white mulberry by analysing gene expression and enzyme activity. in addition, we intend to investigate the stability of the phenolic groups in relation to the organic acid contents of the five different colour types after the drying process, especially since the lighter genotypes and the purple-brown varieties show potential as a dried product. 

So far, only a few recent studies have investigated qualitative and quantitative differences in individual phenolics in mulberry soroses. None of the studies considered five different colour categories to determine the exact chemotype, leading to poor selection criteria from an agronomic point of view. Compared to other horticultural crops, where great progress is being made in breeding new varieties using chemotype markers, research on mulberries is insufficient worldwide. The novelty of this study is that we included 56 local varieties from the existing gene pool of the former Slovenian and Hungarian regions, which we compared with introduced varieties. The results on metabolite composition and size parameters of local and introduced white mulberry varieties could improve their consumption and economic value in Europe. This article underlines the need to preserve mulberries as historical remnants of sericulture in the context of the traditional and rational use of the fruit, as we have found that mulberries are facing dramatic genetic erosion due to the abandonment of sericulture cultivation and agricultural land.

## Figures and Tables

**Table 1:** Average (±SD) stalk length, infructescence length and width for different colour categories of *M. alba* infructescences. Different letters (a–d) indicate significant differences (*p* < 0.001), which were determined using the post hoc Duncan test.

Size Parameters [mm]	Yellowish White	Light Pink	Purple Brown	Reddish Black	Black
Stalk length	6.5 ± 0.1 c	6.2 ± 0.8 c	8.6 ± 0.2 a	7.5 ± 0.2 b	8.2 ± 0.2 ab
Sorosis length	16.6 ± 0.1 c	17.0 ± 0.3 c	21.0 ± 0.3 b	20.8 ± 0.3 b	23.2 ± 0.3 a
Sorosis width	12.0 ± 0.1 d	12.6 ± 0.5 c	13.5 ± 0.1 b	13.1 ± 0.1 c	14.3 ± 0.1 a

**Table 2:** Mean contents (±SE) of sugars and organic acids (mg/100 g FW) in mulberry soroses in relation to colour type. Different letters (a–c) indicate significant differences (*p* < 0.05), which were determined using the post hoc Duncan test.

Sugars	Yellowish White	Light Pink	Purple Brown	Reddish Black	Black
Fructose	2586.60 ± 237.29 ab	2667.90 ± 318.20 a	2503.83 ± 203.87 ab	2246.56 ± 163.10 b	2273.20 ± 142.59 b
Glucose	2384.30 ± 215.00 a	2469.40 ± 203.10 a	2311.60 ± 208.90 a	2084.73 ± 155.65 a	2124.59 ± 133.90 a
Xylose	10.30 ± 2.80 ab	10.64 ± 3.50 a	7.11 ± 1.22 abc	4.95 ± 0.76 bc	4.64 ± 0.69 c
**Organic acids**					
Acetic acid	53.02 ± 24.34 b	9.03 ± 2.30 b	9.03 ± 2.21 b	12.93 ± 4.18 ab	27.75 ± 8.73 a
Citric acid	39.82 ± 4.69 c	66.67 ± 46.09 b	83.57 ± 22.69 b	258.40 ± 61.55 a	257.69 ± 54.75 a
Fumaric acid	24.25 ± 3.22 a	28.45 ± 5.25 a	22.27 ± 2.72 a	10.00 ± 1.94 b	4.80 ± 0.63 b
Lactic acid	16.36 ± 4.36 b	31.88 ± 5.57 ab	19.46 ± 8.25 b	48.35 ± 15.65 a	54.70 ± 17.67 a
Malic acid	92.69 ± 9.50 a	88.60 ± 13.10 a	92.20 ± 11.81 a	90.54 ± 9.52 a	93.01 ± 6.61 a
Succinic acid	81.63 ± 32.64 b	98.26 ± 30.39 ab	159.87 ± 31.12 a	109.31 ± 19.52 ab	92.30 ± 19.12 ab
Tartaric acid	14.07 ± 4.00 b	26.79 ± 3.90 a	17.41 ± 3.02 b	15.75 ± 3.34 b	18.20 ± 3.31 b
**Sugar/acid ratio**	15.48	14.72	11.95	7.95	8.03

**Table 3:** Mean contents (±SE) of individual phenolic compounds (mg/100 g) in different mulberry varieties in relation to the infructescence colour type. Different letters (a–c) indicate significant differences (*p* < 0.05), which were determined using the post hoc Duncan test.

Phenolic acids	Yellowish White	Light Pink	Purple Brown	Reddish Black	Black
**Caffeoylquinic acids**					
3-Caffeoylquinic acid	0.211 ± 0.100 c	1.121 ± 0.091 bc	1.723 ± 0.412 bc	7.435 ± 1.524 ab	11.712 ± 2.021 a
4-Caffeoylquinic acid	3.431 ± 0.475 a	3.878 ± 0.363 a	4.897 ± 0.610 a	5.414 ± 0.707 a	3.671 ± 0.397 a
5-Caffeoylquinic acid 1 (Chlorogenic acid)	6.925 ± 0.846 b	8.232 ± 0.723 b	9.624 ± 1.005 ab	14.366 ± 1.964 a	10.758 ± 0.923 ab
5-Caffeoylquinic acid 2	0.108 ± 0.015 b	0.196 ± 0.031 b	0.294 ± 0.083 ab	0.474 ± 0.07 a	0.303 ± 0.036 ab
Dicaffeoylquinic acid 1	0.052 ± 0.004 c	0.094 ± 0.031 bc	0.185 ± 0.037 a	0.173 ± 0.014 a	0.145 ± 0.017 ab
Dicaffeoylquinic acid 2	0.051 ± 0.005 b	0.098 ± 0.013 a	0.083 ± 0.021 a	0.090 ± 0.011 a	0.141 ± 0.027 a
Dicaffeoylquinic acid 3	0.078 ± 0.013 a	0.081 ± 0.018 a	0.0819 ± 0.036 a	0.093 ± 0.010 a	0.087 ± 0.009 a
**Caffeic acid and its derivatives**					
Caffeic acid	0.176 ± 0.024 a	0.133 ± 0.019 a	0.142 ± 0.034 a	0.147 ± 0.025 a	0.217 ± 0.018 a
Caffeic acid hexoside 1	0.003 ± 0.001 b	0.021 ± 0.016 ab	0.013 ± 0.003 b	0.094 ± 0.018 ab	0.156 ± 0.042 a
Caffeic acid hexoside 2	0.033 ± 0.007 c	0.101 ± 0.014 b	0.172 ± 0.061 b	0.317 ± 0.172 a	0.117 ± 0.036 b
**Coumaroylquinic acids**					
3-*p*-coumaroylquinic acid	0.427 ± 0.053 b	1.312 ± 0.114 ab	2.437 ± 0.542 a	1.971 ± 0.547 a	1.282 ± 0.245 ab
4-*p*-coumaroylquinic acid	0.137 ± 0.024 b	0.222 ± 0.059 ab	0.387 ± 0.148 ab	0.450 ± 0.077 a	0.490 ± 0.069 a
5-Coumaroylquinic acid 1	0.174 ± 0.063 b	0.101 ± 0.024 b	0.309 ± 0.083 a	0.316 ± 0.090 a	0.196 ± 0.057 b
5-Coumaroylquinic acid 2	0.090 ± 0.020 c	0.102 ± 0.017 c	0.171 ± 0.0493 bc	0.351 ± 0.0809 ab	0.492 ± 0.053 a
***p*** **-coumaric acid and its derivatives**					
*p*-coumaric acid	0.006 ± 0.001 a	0.009 ± 0.003 a	0.045 ± 0.018 a	0.167 ± 0.081 a	0.083 ± 0.023 a
*p*-coumaric acid hexoside	0.132 ± 0.027 b	0.178 ± 0.047 b	0.066 ± 0.031 c	0.369 ± 0.020 a	0.189 ± 0.065 b
**Feruloylquinic acids**					
3-Feruloylquinic acid	0.003 ± 0.0001 b	0.008 ± 0.001 ab	0.016 ± 0.001 b	0.030 ± 0.005 a	0.009 ± 0.001 ab
5-Feruloylquinic acid	0.009 ± 0.002 b	0.008 ± 0.001 b	0.009 ± 0.002 b	0.056 ± 0.021 a	0.047 ± 0.018 a
**Protocatechuic acid**	0.143 ± 0.126 a	0.015 ± 0.002 b	0.008 ± 0.001 c	0.009 ± 0.001 c	0.014 ± 0.001 b

**Table 4:** Mean contents (±SE) of individual flavonoids (mg/100 g) in different mulberry varieties in relation to the infructescence colour type. Different letters (a–c) indicate significant differences (*p* < 0.05), which were determined using the post hoc Duncan test.

Flavonoids	Yellowish White	Light Pink	Purple Brown	Reddish Black	Black
**Flavonols**					
**Quercetin derivatives**					
Quercetin-3-galactoside	0.047 ± 0.004 ab	0.038 ± 0.007 b	0.045 ± 0.010 ab	0.067 ± 0.010 ab	0.084 ± 0.010 a
Quercetin-3-glucoside	0.388 ± 0.044 a	0.447 ± 0.094 a	0.312 ± 0.083 a	0.443 ± 0.037 a	0.372 ± 0.024 a
Quercetin-3-rutinoside	1.263 ± 0.144 b	1.383 ± 0.276 b	1.321 ± 0.205 b	2.143 ± 0.201 ab	2.584 ± 0.229 a
Quercetin-3-xyloside	0.001 ± 0.0002 b	0.004 ± 0.001 a	0.002 ± 0.001 b	0.002 ± 0.0003 b	0.003 ± 0.0004 ab
Quercetin-rutinoside hexoside	0.091 ± 0.010 b	0.123 ± 0.019 ab	0.125 ± 0.020 ab	0.203 ± 0.031 a	0.194 ± 0.025 a
Quercetin	0.025 ± 0.007 b	0.029 ± 0.005 ab	0.053 ± 0.009 ab	0.042 ± 0.004 ab	0.058 ± 0.008 a
Quercetin dihexoside	0.078 ± 0.011 b	0.078 ± 0.016 b	0.067 ± 0.010 b	0.131 ± 0.030 a	0.085 ± 0.010 b
Quercetin malonylglucoside	1.126 ± 0.118 ab	1.294 ± 0.187 a	0.807 ± 0.275 b	0.886 ± 0.103 ab	0.654 ± 0.098 b
Quercetin rhamnosyl-hexoside	0.094 ± 0.008 a	0.103 ± 0.009 a	0.097 ± 0.009 a	0.133 ± 0.014 a	0.127 ± 0.014 a
**Kaempferol derivatives**					
Kaempferol hexoside	0.078 ± 0.006 a	0.080 ± 0.022 a	0.019 ± 0.007 c	0.045 ± 0.007 bc	0.063 ± 0.007 ab
Kaempferol-3-rutinoside	0.054 ± 0.007 b	0.073 ± 0.015 ab	0.058 ± 0.013 b	0.079 ± 0.006 a	0.082 ± 0.009 a
**Laricitrin hexoside**	0.016 ± 0.001 bc	0.066 ± 0.016 a	0.009 ± 0.004 c	0.020 ± 0.007 bc	0.046 ± 0.008 ab
**Flavanols**					
Catechin	0.279 ± 0.054 c	0.215 ± 0.031 b	0.382 ± 0.261 b	0.987 ± 0.405 ab	1.099 ± 0.348 a
Epicatechin	0.005 ± 0.001 b	0.003 ± 0.001 b	0.003 ± 0.001 b	0.007 ± 0.001 b	0.014 ± 0.002 a
Procyanidin dimer 1	1.708 ± 0.386 b	0.769 ± 0.230 c	1.121 ± 0.238 b	51.886 ± 16.921 a	114.450 ± 21.416 a
Procyanidin dimer 2	0.466 ± 0.101 b	0.336 ± 0.065 b	0.231 ± 0.077 b	0.488 ± 0.040 b	1.657 ± 0.411 a
**Flavanones**					
**Naringenin derivatives**					
Naringenin hexoside 1	0.012 ± 0.002 c	0.030 ± 0.005 bc	0.028 ± 0.009 bc	0.051 ± 0.007 ab	0.065 ± 0.009 a
Naringenin hexoside 2	0.031 ± 0.003 a	0.014 ± 0.003 b	0.003 ± 0.001 c	0.008 ± 0.001 bc	0.009 ± 0.001 bc
Naringenin hexoside 3	0.007 ± 0.001 a	0.005 ± 0.001 a	0.001 ± 0.0003 b	0.001 ± 0.0001 b	0.001 ± 0.001 b
**Flavons**					
Isoharmnetin hexoside	0.002 ± 0.0002 a	0.004 ± 0.001 bc	0.003 ± 0.001 bc	0.010 ± 0.001 a	0.007 ± 0.001 ab
**Anthocyanins**					
Cyanidin-3-glucoside	0 ± 0 0	2.339 ± 0.817 d	7.921 ± 1.825 c	109.390 ± 25.288 b	165.772 ± 20.666 a
Cyanidin-3-rutinoside	0 ± 0 0	0.242 ± 0.088 b	1.321 ± 0.252 b	17.536 ± 5.703 b	69.901 ± 10.423 a
Pelargonidin-3-glucoside	0 ± 0 0	0.276 ± 0.083 c	0.885 ± 0.201 b	13.083 ± 3.389 a	15.276 ± 1.213 a
**Total phenolics**	231.059 ± 4.625 c	243.166 ± 11.449 c	264.581 ± 11.441 c	364.896 ± 20.398 b	435.249 ± 8.535 a

**Table 5:** Pearson’s correlation coefficient between anthocyanins, sugars, organic acids, and individual phenolics in respect to different infructescence colour types. ** Correlation is significant at the 0.01 level (2-tailed); * Correlation is significant at the 0.05 level (2-tailed).

Biochemical Compounds	Cyanidin-3-Glucoside	Cyanidin-3-Rutinoside	Pelargonidin-3-Glucoside
Xylose	−0.304	−0.253	−0.337 *
Citric acid	0.286	0.172	0.400 *
Fumaric acid	−0.570 **	−0.476 **	−0.611 **
3-Caffeoylquinic acid 1	0.849 **	0.780 **	0.760 **
5-Caffeoylquinic acid 2	0.323 *	0.218	0.359 *
Caffeic acid	0.329 **	0.374 **	0.230
Caffeic acid hexoside1	0.573 **	0.589 **	0.507 **
Caffeic acid hexoside 2	0.230	0.014	0.548 **
4-*p*-Coumaroylquinic acid	0.452 *	0.502 **	0.398
*p*-Coumaric acid	0.456 **	0.448 **	0.419 *
*p*-Coumaric acid hexoside	0.220	0.005	0.535 **
5-Feruloylquinic acid	0.501 **	0.352 *	0.445 **
Quercetin-3-galactoside	0.314 *	0.270 *	0.271 *
Quercetin-3-rutinoside	0.800 **	0.814 **	0.664 **
Quercetin-rutinoside hexoside	0.401 **	0.312 *	0.459 **
Quercetin	0.465 **	0.506 **	0.291
Quercetin dihexoside	0.216 *	0.018	0.420 **
Kaempferol-3-rutinoside	0.315 *	0.336 *	0.373 *
Catechin	0.229	0.151	0.320 *
Epicatechin	0.432 **	0.456 **	0.403 *
Procyanidin dimer 1	0.875 **	0.949 **	0.666 **
Naringenin hexoside 1	0.539 **	0.600 **	0.393 *

## Data Availability

The data used to support the findings of this study can be made available by the corresponding author upon request.

## Supplementary Materials

The following supporting information can be downloaded at: https://www.mdpi.com/article/10.3390/foods12213985/s1, Supplementary Table S1. List of local mulberry genotypes; Supplementary Table S2. The mean concentrations of the sugars and organic acids; Supplementary Table S3. Two-way ANOVA results for the effect of species and soroses’ colour types on different biochemical traits. Table S4. Negative molecular ion mode ([M-H]-) and MS 2 fragmentation data of individual phenolic compounds; Supplementary Table S5. The mean concentrations of the phenolic acids; Supplementary Table S6. The mean concentrations of the flavonoids; Supplementary Table S7. Pooled within-group correlations between variables and PC functions of the PCA diagram.
