# Supplementary material for: The Darker the Better: Identification of Chemotype Profile in Soroses of Local and Introduced Mulberry Varieties with Respect to the Colour Type
Source: Foods. 2023 Oct 31;12(21):3985. doi: 10.3390/foods12213985 (PMC10650418; doi:10.3390/foods12213985)
Supplement: Supplementary file 1 [file foods-12-03985-s001.zip › foods-2686835-supplementary.pdf]

Supplementary Table S1. List of local mulberry genotypes form Slovenia and Hungary planted in the mulberry collection of the Faculty of Agriculture University Maribor with location of their origin, geographic coordinates and detailed data along with the list and specification of reference sericultural and fruit varieties. The identification No. is defined by the abbreviation of the region (Slovenia) or county (Hungary). Slovenia: SE- Southeastern region, SM- Submediterranean region, SP- Subpannonean region; Hungary: BA-Baranya, BE- Bekes, GMS- Györ-Moson-Sopron, PE- Pest, SO-Somogy, TO-Tolna, VE-Veszprem ,ZA-Zala;

Group name/Ident. No.	Colour	Region	Detailed regionalization	Abb.	Detailed location	Samplingd ate	Lat. (° N)	Long. (° E)	CBH (cm)
**Slovenian genotypes**									
SE 19.2	LP	Southeastern	Krška raven	SE	Brege	13.7.2016	45.921120	15.49817	152
SE 9.1	B	Southeastern	Črnomelj	SE	Marindol	6.7.2016	45.506130	15.328900	190
SE 9.2	B	Southeastern	Črnomelj	SE	Marindol	6.7.2016	45.506130	15.328900	190
SM 208	YW	Submediterranean	Koprska brda	SM	Abitanti	29.6.2016	45.436550	13.825140	130
SP 300	YW	Subpannonean	Dravska ravan	SP	Rošnja	8.6.2017	46.48013	15.74335	276
SP 256	B	Subpannonean	Lendava	SP	Dobrovnik	26.7.2016	46.655350	16.354410	178
SP 272	RB	Subpannonean	Goričko	SP	Bodonci	26.7.2016	46.744130	16.090650	178
SP 306	B	Subpannonean	Haloze	SP	Žetale	8.6.2017	46.273440	15.791595	160
SP 249	YW	Subpannonean	Murska ravan	SP	Stara nova vas	20.7.2016	46.582110	16.117470	70
SP 8	B	Subpannonean	Murska ravan	SP	Stogovci	20.7.2016	46.694120	15.834570	172
SE 24	RB	Southeastern	Novomeška	SE	Ostrog	13.7.2016	45.856240	15.37738	275
SE 290	B	Southeastern	Metlika	SE	Vinomer-Drasici	2.8.2016	45.669010	15.364560	190
SE 5	YW	Southeastern	Črnomelj	SE	Castle Dragatus	6.7.2016	45.520700	15.159010	122
SM 100.1	YW	Submediterranean	Kras	SM	Castle Školj	23.5.2017	45.66090	14.00696	189
SM 214	YW	Submediterranean	Goriška brda	SM	Medana .	4.7.2016	45.982020	13.519870	120
SM 29	RB	Submediterranean	Vipavska	SM	Potoče	27.5.2015	45.88809	13.821810	/
SP 10	YW	Subpannonean	Sloven. gorice	SP	Dražen vrh	20.7.2016	46.65360	15.799760	270
SP 237	RB	Subpannonean	Sloven. gorice	SP	Zenik	20.7.2016	46.556780	16.012760	145
**Hungarian genotypes**									
SO 1035	LP	South Transdanubia	Somogy	SO	Mernye	30.5.2017	46.516639	17.819101	2x65
BA 2111	B	South Transdanubia	Baranya	BA	Szuliman	14.6.2017	46.123830	17.819420	265
BA 2126	YW	South Transdanubia	Baranya	BA	Ketujfalu	14.6.2017	45.981790	17.732420	170

BA 2179	B	South Transdanubia	Baranya	BA	Dunaszekso	14.6.2017	46.066690	18.746480	/
BE 1264.2	PB	Southern Great Plain	Bekes	BE	Bekescsaba	5.7.2017	46.684300	21.087600	182
GMS 2357	YW	Western Transdanubia	Gyor-Moson-Sopron	HU	Morichida	20.6.2017	47.517870	17.412390	236
PE 4	YW	Central Hungary	Pest	PE	Nosziop	29.11.2014	47.183340	17.458510	
PE 61214	RB	Central Hungary	Pest	PE	Budapest	6.12.2014	/	/	/
SO 2008	PB	South Transdanubia	Somogy	SO	Csurgo	30.5.2017	46.247080	17.105960	120
SO 2018	PB	South Transdanubia	Somogy	SO	Barcs	30.5.2017	45.985240	17.426230	310
VA 1051	B	Western Transdanubia	Vas	VA	Apatisvanfalva	6.6.2017	46.942500	16.274500	142
VA 1056	RB	Western Transdanubia	Vas	VA	Alsojanoshegyl	6.6.2017	46.886200	16.170000	/
VA 2570	LP	Western Transdanubia	Vas	VA	Sitke	20.6.2017	47.243470	17.023680	220
VE 2620	YW	Central Transdanubia	Veszprem	VE	Sumeg	27.6.2017	46.980800	17.290600	170
ZA 2041	B	Western Transdanubia	Zala	ZA	Tornyiszentmik	6.6.2017	46.529500	16.559300	210
ZA 2044	YW	Western Transdanubia	Zala	ZA	Szecsisziget	6.6.2017	46.592800	16.615050	60
ZA 2045	RB	Western Transdanubia	Zala	ZA	Paka	6.6.2017	46.593750	16.648510	205
ZA 2047	PB	Western Transdanubia	Zala	ZA	Radihaza	6.6.2017	46.649450	16.780080	110
ZA 2053	RB	Western Transdanubia	Zala	ZA	Sojtor. Deak	6.6.2017	46.685670	16.856180	130
ZA 2084	B	Western Transdanubia	Zala	ZA	Kiliman - Gelse	6.6.2017	46.628430	16.995460	60
ZA 2095	RB	Western Transdanubia	Zala	ZA	Szentmargitfalv	6.6.2017	46.494230	16.656630	190
**Sericultural varieties**	**Colour**	**Origin**	**Obtained**			**Prop./**	**planting**	**date**	
Morus alba (L.) 'Florio'	PB	Italy	mulberry gene bank CREA Padua, Italy			3.2015/10.2015			
M. alba (L.) 'Kokusou'	B	Japan	mulberry gene bank CREA Padua, Italy			3.2015/10.2017			
M. alba (L.) 'Morettiana'	YW	Italy	mulberry gene bank CREA Padua, Italy			3.2015/10.2018			
M. alba(L.) 'Muki'	B	/	mulberry gene bank CREA Padua, Italy			3.2015/10.2018			
**Fruit varieties**									
M. alba 'Agathe'	RB	/		Rabensteiner J.		23.6.2018			

M. alba 'Red'	RB	/	Rabensteiner J.	23.6.2018
M. alba 'White'	PB	/	Hubmann (from a collector in Bulgaria)	/
M. alba 'Шелл № 150'	PB	Hybrid from Ukraine. Poltawa (Schell 150)	/	/
M. alba s.l. 'Big Ten'	B	/	Pucher	/
M. alba s.l. 'Black'	B	Hybrid from Romania	Hubmann (collector in Bulgaria)	/
M. alba s.l. 'CREA fruit selection'	B	/	mulberry gene bank CREA Padua. Italy	1.3.2015
M. alba s.l. 'Yellow Roso'	B	/	Hubman	2014/3.2016
M. indica 'Coree'	B	Variety from France	Hubmann (originally from a collector in Turkey)	/
M. indica 'Shin-Tso'	B	/	Hubmann (originally from cornusmas.eu)	/
M. alba × *rubra*	RB	Hybrid	Hubmann. Austria (original from tree in Graz)	2014/3.2016
M. alba x *M. rubra* 'Illinois Everbearing'	RB	Unknown	Pucher	/
M. alba x *M. rubra* 'Ivory'	B	Hybrid from Canada	Hubmann (original introduced via hortensis.de)	/
M. alba x *M. rubra* ′Frech Hybrid'	RB	Hybrid from France	Pucher (original http://www.cochetfrederic.com)	/
M. nigra	B	Local genotype. East Styria. Austria	Pucher, Austria. lat.46.97057256490299, long. 15.70354170496526	2014/3.2016

Supplementary Table S2: The mean concentrations of the sugars and organic acids (mg/100 g FW) in soroses of Slovenian, Hungarian old mulberry genotypes, sericultural and fruit varieties.

Group name/Identification No.	Glucose	Xylose	Fructose	Citric acid	Tartaric acid	Malic acid	Succinic acid	Lactic acid	Fumaric acid	Acetic acid
**Slovenian mulberry genotypes**										
SE 5	2018.62	2.63	2218.52	38.54	0.00	66.17	4.31	12.92	22.24	1.08
SE 9.1	1544.71	1.58	1673.90	138.49	26.86	76.76	108.91	8.35	4.52	0.00
SE 9.2	1930.79	0.00	2025.65	68.87	29.99	95.07	108.01	0.00	11.25	5.24
SE 19.2	2463.59	5.17	2689.97	20.57	22.89	103.15	153.55	41.39	25.75	3.25
SE 24	1795.05	7.39	1944.13	61.81	22.95	74.64	143.10	11.60	14.41	4.92
SE 290	1939.38	1.91	2146.87	88.80	16.45	91.16	136.56	8.43	8.07	6.89
SM 29	1528.43	0.00	1640.71	35.05	17.17	75.37	166.95	15.36	23.08	4.47
SM 100.1	3799.46	15.22	4019.71	0.00	0.00	112.66	10.60	14.87	37.57	0.00
SM 208	2691.46	33.82	2908.13	48.05	10.05	87.83	384.74	11.95	31.74	4.07
SM 214	2593.72	13.05	2694.87	0.00	0.00	104.98	10.11	32.93	24.10	294.04
SP 8	2562.88	6.18	2757.85	97.73	17.69	105.44	189.94	9.66	7.44	7.18
SP 10	2807.91	7.48	3092.26	27.10	22.07	151.54	225.62	8.55	16.66	9.00
SP 237	1198.00	0.00	1251.34	37.89	16.76	69.26	198.17	5.81	10.12	5.18
SP 249	1023.02	1.93	1117.22	0.00	0.00	48.27	23.36	3.79	5.39	18.16
SP 256	1236.73	2.95	1307.16	85.01	8.92	69.50	115.85	7.63	6.00	1.94
SP 272	1744.40	4.61	1925.36	185.86	0.00	66.47	5.30	0.00	10.97	5.58
SP 300	2285.83	13.75	2495.02	0.00	0.00	78.72	9.09	10.72	29.36	123.32
SP 306	1289.79	3.88	1452.17	0.00	0.00	73.28	3.99	14.73	1.44	34.24
**Hungarian mulberry genotypes**										
BA 2111	1499.59	0.00	1561.71	0.00	0.00	47.23	8.81	0.00	0.58	23.69
BA 2126	3118.24	11.68	3549.88	0.00	0.00	119.75	21.44	62.83	17.17	41.37
BA 2179	2565.76	4.69	2788.18	180.67	18.04	123.27	141.42	85.83	5.24	4.62
BE 1264.2	1830.04	7.79	2131.42	0.00	0.00	72.38	14.92	1.43	12.04	16.53

GMS 2357	1835.75	0.00	1998.16	0.00	0.00	71.73	21.95	4.32	21.83	16.31
PE 4	2807.47	4.97	2962.91	45.58	10.09	147.49	210.42	17.17	25.46	3.84
PE 61214	1671.73	5.22	1820.46	102.56	20.15	64.48	123.09	2.43	2.28	7.82
SO 1035	2793.15	13.20	3002.85	0.00	0.00	84.47	8.56	16.01	30.17	10.96
SO 2008	1846.33	12.93	2069.26	41.18	20.98	61.73	139.76	16.90	25.33	6.46
SO 2018	1552.48	4.91	1688.82	39.69	10.67	91.75	134.95	0.22	18.38	4.61
VA 1051	1583.30	0.00	1687.67	68.92	19.01	83.78	61.96	0.44	6.57	3.91
VA 1056	1918.27	2.11	2186.73	0.00	0.00	85.74	12.30	26.70	24.84	53.14
VA 2570	1503.72	8.43	1641.00	112.76	30.70	86.43	312.10	38.07	25.99	0.00
VE 2620	2588.49	7.21	2834.23	0.00	0.00	99.18	32.69	13.34	40.53	82.35
ZA 2041	0.00	0.00	0.00	104.34	12.33	72.49	120.89	12.49	12.30	2.12
ZA 2044	883.84	1.83	988.54	0.00	0.00	41.32	76.51	8.17	4.22	30.88
ZA 2045	2137.28	7.07	2313.41	121.50	16.97	80.97	209.80	24.12	9.01	8.75
ZA 2047	2431.70	7.76	2668.71	99.57	24.25	132.70	413.52	7.13	20.71	14.52
ZA 2053	2871.91	5.65	3088.68	173.18	20.93	120.01	168.46	7.40	1.57	2.66
ZA2084	2047.92	9.55	2288.07	0.00	0.00	71.36	2.76	1.16	4.14	30.59
ZA2095	1093.67	9.62	1244.89	442.32	45.71	45.88	73.25	14.45	9.92	0.00
**Reference sericultural varieties**										
Morus alba (L.) 'Florio'	3149.85	7.70	3266.78	51.75	21.47	109.41	219.47	57.84	26.60	8.38
M. alba (L.) 'Kokusou'	1542.85	0.00	1608.73	384.94	0.49	132.60	83.79	218.29	6.80	14.27
M. alba (L.) 'Morettiana'	2542.33	0.00	2746.32	0.00	0.00	75.35	30.30	11.07	38.93	11.83
M. alba (L.) 'Muki'	2400.94	0.00	2569.92	115.76	33.60	135.54	361.83	295.16	0.11	11.34
**Fruit varieties**										
M. alba (L.) 'Agathe'	1330.07	4.35	1428.17	138.88	14.91	85.61	230.38	61.12	14.96	2.56
M. alba (L.) 'Red'	2794.77	0.00	2935.47	627.15	17.54	148.65	213.70	80.47	4.02	45.50
M. alba (L.) 'White'	1768.83	2.22	1846.24	60.05	9.56	106.57	146.83	10.72	30.65	2.47
M. alba (L.) M 150/N 01 'Шелл № 150'	1458.87	2.84	1556.55	165.31	10.77	114.15	166.89	22.76	18.27	5.80
M. alba (L.) s.l. 'Big Ten'	1713.43	0.81	1875.69	137.33	0.00	111.08	1.56	0.00	2.56	54.29

M. alba (L.) s.l. 'Black'	1963.92	6.96	2083.23	380.60	27.38	90.88	72.92	106.29	4.72	10.35
M. alba (L.) s.l. 'CREA fruit selection'	1876.15	3.07	1959.48	159.69	0.00	42.98	1.75	3.34	3.24	15.42
M. alba (L.) s.l. 'Yellow roso'	3061.22	1.60	3178.50	175.37	0.10	139.69	279.28	16.93	9.21	5.02
M. indica (L.) 'Coree'	2819.16	3.46	2984.25	445.27	0.00	155.56	139.51	76.42	4.36	10.41
M. indica (L.) 'Shin-Tso'	3756.17	10.24	4088.94	352.48	0.00	74.52	2.10	0.00	2.51	17.26
M. alba × *rubra*	2949.34	0.87	3179.71	557.24	0.21	182.86	47.70	192.37	0.66	7.03
M. alba x *M. rubra* 'Illinois Everbearing'	2616.13	0.00	2694.87	613.53	0.08	123.98	68.92	74.34	2.60	3.82
M. alba x *M. rubra* 'Ivory'	2624.42	2.23	2782.96	404.95	3.76	143.06	113.73	70.05	5.54	52.51
M. alba x *M. rubra* French hybrid	2707.29	3.79	2882.79	480.95	0.68	135.40	43.25	175.16	0.77	8.36
M. nigra (L.)	1747.28	4.14	1925.96	1058.76	0.00	71.28	3.56	0.00	0.71	132.44

Supplementary Table S3: Two way ANOVA results for the effect of species and soroses colour types on different biochemical traits.

	df	F	Sig.
**Fructose**			
species	3	4.55	**0.006**
colour	4	2.39	0.061
species * colour	1	0.28	0.597
**Glucose**			
species	3	4.49	**0.007**
colour	4	1.79	0.145
species * colour	1	0.81	0.371
**Xylose**			
species	3	0.10	0.962
colour	4	3.23	**0.018**
species * colour	1	0.69	0.409
**Citric acid**			
species	3	16.60	**<0.001**
colour	4	3.79	**0.008**
species * colour	1	5.90	**0.018**
**Fumaric acid**			
species	3	0.75	0.528
colour	4	15.6	**<0.001**
colour * species	1	1.08	0.304
**Tartaric acid**			
species	3	3.65	**0.018**
colour	4	2.42	0.059
species * colour	1	0.13	0.725

	df	F	Sig.
**Quercetin-rutinoside hexoside**			
species	3	0.53	0.667
colour	4	3.30	**0.017**
species * colour	1	11.1	**0.001**
**Quercetin dihexoside**			
species	3	8.58	**<0.001**
colour	4	5.28	**0.001**
species * colour	1	24.8	**<0.001**
**Kaempferol hexoside**			
species	3	3.82	**0.014**
colour	4	6.25	**<0.001**
species * colour	1	4.83	**0.032**
**Kaempferol-3-rutinoside**			
species	3	0.73	0.537
colour	4	2.40	0.06
species * colour	1	5.58	**0.021**
**Laricitrin hexoside**			
species	3	2.91	**0.042**
colour	4	3.54	**0.012**
species * colour	1	0.73	0.395
**Epicatechin**			
species	3	2.54	0.065
colour	4	4.05	**0.006**
species * colour	1	3.13	0.082

**5-Caffeoylquinic acid 1 (Chlorogenic acid)**			
species	3	0.16	0.921
colour	4	4.64	**0.003**
species * colour	1	6.94	**0.011**
**Caffeoylquinic acids**			
species	3	3.28	**0.029**
colour	4	5.55	**<0.001**
species * colour	1	0.11	0.741
**Caffeic acid derivatives**			
species	3	8.36	**<0.001**
colour	4	1.04	0.397
species * colour	1	4.96	**0.031**
**Coumaroylquinic acids**			
species	3	5.24	**0.003**
colour	4	3.52	**0.013**
species * colour	1	2.84	0.098
**p-coumaric acid derivatives**			
species	3	6.44	**<0.001**
colour	4	2.81	**0.035**
species * colour	1	11.40	**0.001**
**Feruloylquinic acids**			
species	3	3.93	**0.014**
colour	4	1.18	0.332
species * colour	1	0.11	0.743
**Quercetin-3-galactoside**			
species	3	0.37	0.773
colour	4	1.30	0.279

**Procyanidin dimer 1**			
species	3	2.71	0.053
colour	4	7.25	**<0.001**
species * colour	1	2.07	0.155
**Naringenin hexoside 1**			
species	3	2.62	0.059
colour	4	6.42	**<0.001**
species * colour	1	0.02	0.896
**Naringenin hexoside 2**			
species	3	1.29	0.286
colour	4	26.40	**<0.001**
species * colour	1	0.24	0.623
**Naringenin hexoside 3**			
species	3	0.54	0.657
colour	4	19.2	**<0.001**
species * colour	1	0.01	0.958
**Isoharmnetin hexoside**			
species	3	3.73	**0.016**
colour	4	3.60	**0.011**
species * colour	1	1.94	0.169
**Cyanidin-3-glucoside**			
species	3	6.78	**<0.001**
colour	4	14.30	**<0.001**
species * colour	1	4.40	**0.040**
**Cyanidin-3-rutinoside**			
species	3	3.09	**0.034**
colour	4	10.30	**<0.001**

species * colour	1	5.29	**0.026**
**Quercetin-3-rutinoside**			
species	3	4.37	**0.008**
colour	4	8.19	**<0.001**
species * colour	1	0.01	0.928
**Quercetin-3-xyloside**			
species	3	2.12	0.107
colour	4	3.13	**0.021**
species * colour	1	6.05	**0.017**

species * colour	1	3.69	0.060
**Pelargonidin-3-glucoside**			
species	3	4.02	**0.011**
colour	4	10.50	**<0.001**
species * colour	1	1.73	0.194
**Total phenolics**			
species	3	1.52	0.220
colour	4	30.10	**<0.001**
species * colour	1	1.92	0.171

Supplementary Table S4. Negative molecular ion mode ([M-H] -) and MS 2 fragmentation data of individual phenolic compounds in mulberry leaves detected by HPLC-MS.

**Caffeoylquinic acids**	**$[MH]^+$ or $[MH]^-$**	**MS^2**
3-Caffeoylquinic acid	353	191, 179, 135
4-Caffeoylquinic acid	353	173, 179, 191
5-Caffeoylquinic acid 1 (chlorogenic acid)	353	191, 179
5-Caffeoylquinic acid 2	353	191, 179
Dicaffeoylquinic acid 1	515	353, 191,. 179, 173
Dicaffeoylquinic acid 2	515	353, 191, 173
Dicaffeoylquinic acid 3	515	353, 173, 179, 191
**Caffeic acid and its derivatives**		
Caffeic acid	179	135
Caffeic acid hexoside 1	341	179, 161
Caffeic acid hexoside 2	341	179, 161
**Coumaroylquinic acids**		
3-*p*-Coumaroylquinic acid	337	163, 191, 173
4-*p*-Coumaroylquinic acid	337	173, 163, 191
5-*p*-Coumaroylquinic acid 1	337	191, 173, 163
5-*p*-Coumaroylquinic acid 2	337	191, 173, 163
***p*-coumaric acid and its derivatives**		
p-Coumaric acid	163	119
p-Coumaric acid hexoside	325	163
**Feruloylquinic acids**		
3-Feruloylquinic acid	367	193, 134
5-Feruloylquinic acid	367	193, 191, 173
**Protocatechuic acid**	153	109
**Flavonoids**		
**Flavonols**		

**Quercetin derivatives**		
Quercetin-3-galactoside	463	301
Quercetin-3-glucoside	463	301
Quercetin-3-rutinoside	609	301
Quercetin-3-xyloside	433	301
Quercetin-rutinoside hexoside	771	301
Quercetin	301	179, 151
Quercetin dihexoside	625	301
Quercetin malonylglucoside	549	463, 301
Quercetin rhamnosylhexoside	755	609, 301
**Kaempferol derivatives**		
Kaempferol hexoside	447	285
Kaempferol-3-rutinoside	593	447, 285
Laricitrin hexoside	493	331
**Flavanols**		
Catechin	289	245
Epicatechin	289	245
Procyanidin dimer 1	577	451, 425, 407, 289
Procyanidin dimer 2	577	451, 425, 407, 289
**Flavanons**		
Naringenin derivatives		
Naringenin hexoside 1	433	271
Naringenin hexoside 2	433	271
Naringenin hexoside 3	433	271
**Flavones**		
Isorhamnetin hexoside	477	315
**Anthocyanins**		
Cyanidin-3-glucoside	449	287

Cyanidin-3-rutinoside	595	449, 287
Cyanidin-3-sophoroside	611	287, 181, 153
Pelargonidin-3-glucoside	433	271
Pelargonidin-3-rutinoside	579	271, 215, 153, 91
Peonidin-3-rutinoside	609	301
Petunidin-3-glucoside	479	317

Supplementary Table S5: The mean concentrations of the total phenolics and phenolic acids (mg/100 g FW) in soroses of Slovenian, Hungarian old mulberry genotypes, sericultural and fruit varieties.

Group name/Identification No.	Total phenolics	Caffeoylquinic acids	Caffeic a. derivatives	Coumaroylquinic acids	*p*-coumaric a. der.	Feruloylquinic acids	protocatechuic a.
**Slovenian mulberry genotypes**							
SE 5	217.450	6.088	0.179	0.703	0.204	0.019	0.016
SE 9.1.	436.853	25.949	0.290	1.522	0.071	0.030	0.012
SE 9.2.	438.730	18.855	0.216	1.149	0.125	0.016	0.013
SE 19.2	253.708	14.715	0.267	1.760	0.284	0.019	0.016
SE 24	268.156	16.823	0.152	1.103	0.038	0.032	0.010
SE 290	450.273	20.563	0.232	1.756	0.116	0.027	0.013
SM 29	291.208	15.303	0.198	1.148	0.023	0.029	0.007
SM 100.1	251.310	10.822	0.286	0.582	0.239	0.006	0.017
SM 208	231.360	15.436	0.136	0.934	0.044	0.012	0.015
SM 214	253.013	16.472	0.300	1.853	0.015	0.034	0.015
SP 8	433.005	20.925	0.238	1.636	0.050	0.028	0.013
SP 10	233.068	13.032	0.188	0.864	0.264	0.016	0.018
SP 237	256.930	10.609	0.076	0.995	0.006	0.020	0.008
SP 249	220.393	8.407	0.124	0.495	0.009	0.005	1.660
SP 256	500.765	30.997	0.446	1.691	0.210	0.020	0.014
SP 272	353.865	18.013	0.199	1.229	0.082	0.031	0.008
SP 300	232.390	17.824	0.262	0.964	0.260	0.016	0.015
SP 306	354.868	29.489	0.323	2.454	0.076	0.041	0.012
**Hungarian mulberry genotypes**							
BA 2111	456.500	43.893	0.449	2.883	0.007	0.035	0.013
BA 2126	210.245	4.173	0.201	0.478	0.178	0.005	0.020
BA 2179	401.195	12.138	0.156	0.881	0.023	0.009	0.012

BE 1264.2	308.330	23.665	0.844	3.610	0.100	0.034	0.007
GMS 2357	230.888	11.724	0.368	0.639	0.177	0.010	0.017
PE 4	221.785	10.937	0.092	0.766	0.023	0.009	0.017
PE 61214	463.925	26.682	0.476	1.504	0.707	0.051	0.003
SO 1035	242.804	12.803	0.245	1.760	0.162	0.016	0.013
SO 2008	243.820	18.279	0.203	2.002	0.010	0.023	0.006
SO 2018	236.175	8.134	0.120	0.813	0.013	0.018	0.008
VA 1051	401.653	22.504	0.318	1.426	0.122	0.022	0.012
VA 1056	331.830	15.461	0.179	1.298	0.010	0.036	0.011
VA 2570	233.710	15.510	0.281	1.645	0.141	0.013	0.017
VE 2620	205.250	2.571	0.085	0.644	0.119	0.003	0.017
ZA 2041	446.983	33.507	0.406	1.423	0.501	0.020	0.012
ZA 2044	234.875	15.657	0.326	0.792	0.033	0.015	0.015
ZA 2045	288.335	12.914	0.127	1.100	0.011	0.025	0.008
ZA 2047	220.525	13.963	0.190	1.908	0.019	0.021	0.006
ZA 2053	442.708	35.449	0.574	2.751	0.592	0.072	0.008
ZA 2084	438.235	18.377	0.339	1.673	0.180	0.026	0.013
ZA 2095	472.955	52.677	0.686	3.285	0.092	0.168	0.003
**Reference sericultural varieties**							
Morus alba (L.) 'Florio'	281.280	21.403	0.205	2.588	0.061	0.033	0.007
M. alba (L.) 'Kokusou'	408.390	29.543	0.363	1.833	0.083	0.030	0.012
M. alba (L.) 'Morettiana'	261.738	7.512	0.189	0.448	0.096	0.010	0.022
M. alba (L.) 'Muki'	476.863	20.227	0.314	0.763	0.072	0.016	0.014
**Fruit varieties**							
M. alba (L.) 'Agathe'	288.718	25.592	0.441	5.169	0.218	0.062	0.026
M. alba (L.) 'Red'	389.373	35.375	0.126	5.205	0.054	0.153	0.009
M. alba (L.) 'White'	290.850	14.077	0.168	1.599	0.155	0.032	0.007
M. alba (L.) M 150/N 01 'Шелл № 150'	253.630	13.413	0.346	5.364	0.195	0.016	0.011

M. alba (L.) s.l. 'Big Ten'	479.645	31.979	0.726	5.333	0.338	0.377	0.005
M. alba (L.) s.l. 'Black'	397.946	25.894	0.325	2.324	0.643	0.016	0.008
M. alba (L.) s.l. 'CREA fruit selection'	471.639	45.201	1.031	3.366	0.187	0.023	0.029
M. alba (L.) s.l. 'Yellow roso'	447.621	27.639	0.545	1.533	0.156	0.020	0.028
M. indica (L.) 'Coree'	378.274	14.689	0.257	4.106	0.108	0.127	0.005
M. indica (L.) 'Shin-Tso'	536.380	12.086	0.375	3.915	0.076	0.037	0.010
M. alba × rubra	447.643	54.962	0.944	8.716	0.906	0.256	0.009
M. alba x *M. rubra* 'Illinois Everbearing'	449.273	26.014	3.309	1.761	3.978	0.132	0.009
M. alba x *M. rubra* 'Ivory'	435.048	39.993	0.856	2.442	0.215	0.170	0.017
M. alba x *M. rubra* 'French hybrid'	428.530	42.934	0.789	6.005	0.722	0.105	0.008
M. nigra (L.)	395.073	17.690	0.844	3.767	0.687	0.007	0.005

Supplementary Table S6: The mean concentrations of the flavonoids (mg/100 g FW) in soroses of Slovenian, Hungarian old mulberry genotypes, sericultural and fruit varieties.

Group name/Identification No.	Quercetin derivatives	Kaempferol derivatives	Laricitrin hexoside	Flavanols	Flavanones	Flavones	Anthocyanins
**Slovenian mulberry genotypes**							
SE 5	3.320	0.130	0.017	4.082	0.0512	0.002	0.000
SE 9.2.	4.414	0.119	0.079	75.984	0.102	0.005	171.247
SE 9.1.	4.393	0.165	0.104	54.172	0.087	0.005	157.059
SE 19.2	4.532	0.250	0.072	1.366	0.066	0.006	3.749
SE 24	3.084	0.116	0.042	38.403	0.059	0.007	42.840
SE 290	3.678	0.099	0.101	59.609	0.075	0.004	193.652
SM 29	4.162	0.088	0.009	1.219	0.088	0.015	45.915
SM 100.1	3.224	0.113	0.017	6.192	0.055	0.002	0.000
SM 208	3.270	0.189	0.008	1.325	0.039	0.002	0.000
SM 214	4.678	0.173	0.025	0.522	0.075	0.003	0.000
SP 8	3.950	0.164	0.099	11.049	0.081	0.005	118.709
SP 10	3.262	0.102	0.016	2.058	0.051	0.002	0.000
SP 237	3.298	0.142	0.009	1.398	0.062	0.016	15.823
SP 249	2.240	0.092	0.013	2.585	0.038	0.001	0.000
SP 256	4.830	0.170	0.058	293.181	0.127	0.005	483.511
SP 272	4.619	0.086	0.013	4.880	0.090	0.016	42.427
SP 300	4.320	0.194	0.019	2.168	0.068	0.003	0.000
SP 306	3.987	0.161	0.106	29.837	0.084	0.004	113.782
**Hungarian mulberry genotypes**							
BA 2111	5.107	0.119	0.115	126.798	0.118	0.005	293.735
BA 2126	1.702	0.098	0.009	2.946	0.028	0.001	0.000
BA 2179	5.090	0.222	0.073	44.164	0.098	0.006	131.322

BE 1264.2	6.278	0.147	0.033	1.105	0.078	0.008	5.330
GMS 2357	2.377	0.076	0.017	2.338	0.046	0.001	0.000
PE 4	3.338	0.190	0.013	1.886	0.048	0.002	0.000
PE 61214	4.664	0.160	0.002	190.944	0.082	0.002	371.013
SO 1035	3.585	0.145	0.067	1.548	0.051	0.004	18.801
SO 2008	2.787	0.084	0.011	1.236	0.039	0.003	13.830
SO 2018	3.954	0.096	0.013	1.309	0.071	0.017	8.816
VA 1051	3.565	0.165	0.066	63.272	0.079	0.004	165.716
VA 1056	1.676	0.032	0.007	0.433	0.029	0.005	9.590
VA 2570	2.189	0.082	0.060	0.393	0.031	0.002	3.751
VE 2620	1.431	0.076	0.010	1.469	0.027	0.001	0.000
ZA 2041	4.521	0.121	0.031	272.166	0.123	0.004	526.283
ZA 2044	4.604	0.202	0.027	0.247	0.084	0.004	0.000
ZA 2045	2.372	0.050	0.008	7.908	0.044	0.008	41.366
ZA 2047	2.651	0.058	0.009	0.455	0.044	0.003	2.178
ZA 2053	6.636	0.171	0.065	163.955	0.123	0.005	315.250
ZA 2084	4.967	0.226	0.099	53.468	0.105	0.005	228.300
ZA 2095	4.903	0.128	0.002	102.683	0.075	0.002	349.878
**Reference sericultural varieties**							
Morus alba 'Florio'	2.123	0.041	0.005	1.512	0.040	0.002	9.668
M. alba 'Kokusou'	5.765	0.260	0.097	62.855	0.125	0.006	168.818
M. alba 'Morettiana'	2.555	0.093	0.028	1.423	0.049	0.004	0.000
M. alba 'Muki'	4.203	0.113	0.066	85.634	0.096	0.004	158.007
**Fruit varieties**							
M. alba × rubra	4.291	0.165	0.016	81.070	0.036	0.013	208.767
M. alba (L.) 'Agathe'	2.951	0.119	0.004	8.036	0.016	0.004	36.823
M. alba (L.) 'Red'	2.411	0.066	0.023	4.833	0.034	0.006	50.181
M. alba (L.) 'White'	2.094	0.056	0.003	2.183	0.013	0.004	6.926

M. alba (L.) M 150/N 01 'Шелл № 150'	1.854	0.082	0.003	2.648	0.006	0.002	15.298
M. alba (L.) s.l. 'Big Ten'	8.178	0.170	0.001	370.840	0.227	0.033	624.259
M. alba (L.) s.l. 'Black'	3.147	0.120	0.003	154.230	0.034	0.009	159.286
M. alba (L.) s.l. 'CREA fruit selection'	4.809	0.264	0.006	252.422	0.026	0.006	390.851
M. indica (L.) 'Shin-Tso'	0.552	0.015	9.53E-05	22.218	0.014	0.004	103.637
M. alba (L.) s.l. 'Yellow roso'	4.145	0.184	0.008	52.543	0.026	0.006	202.123
M. indica (L.) 'Coree'	2.494	0.010	0.001	16.677	0.052	0.018	95.778
M. alba x *M. rubra* 'Illinois Everbearing'	4.124	0.190	0.008	39.518	0.030	0.014	280.647
M. alba x *M. rubra* 'Ivory'	3.738	0.063	0.012	180.200	0.035	0.004	438.129
M. alba x *M. rubra* French hybrid	5.093	0.194	0.019	27.664	0.039	0.019	225.641
M. nigra (L.)	1.526	0.033	6.91E-05	99.549	0.065	0.012	200.047

Supplementary Table S7. Pooled within-group correlations between variables and PC functions of the PCA diagram (see Fig. 2).

	**PC 1**	**PC 2**
Fructose	-0.157	-0.055
Glucose	-0.162	-0.055
Xylose	-0.237	-0.027
Acetic acid	-0.051	0.438
Citric acid	0.336	-0.020
Fumaric acid	-0.378	-0.063
Lactic acid	0.216	0.080
Malic acid	-0.023	0.017
Succinic acid	-0.006	-0.159
Tartaric acid	0.084	-0.350
3-Caffeoylquinic acid	0.133	0.059
4-Caffeoylquinic acid	0.031	-0.154
5-Caffeoylquinic acid 1 (Chlorogenic acid)	0.091	-0.091
5-Caffeoylquinic acid 2	0.106	-0.149
Dicaffeoylquinic acid 1	0.100	-0.246
Dicaffeoylquinic acid 2	0.055	-0.001
Dicaffeoylquinic acid 3	-0.007	0.004
Caffeic acid	0.041	0.132
Caffeic acid hexoside 1	0.095	0.055
Caffeic acid hexoside 2	0.031	-0.089
3-*p*-coumaroylquinic acid	0.040	-0.228
4-*p*-coumaroylquinic acid	0.100	-0.073
5-*p*-Coumaroylquinic acid 1	0.020	-0.089
5-*p*-Coumaroylquinic acid 2	0.136	0.043
p-coumaric acid	0.056	-0.042
p-coumaric acid hexoside	0.023	-0.009
3-Feruloylquinic acid	0.084	-0.214
5-Feruloylquinic acid	0.068	0.017
Protocatechuic acid	-0.038	0.102
Quercetin-3-galactoside	0.087	0.101
Quercetin-3-glucoside	0.001	0.038
Quercetin-3-rutinoside	0.126	0.070
Quercetin-3-xyloside	0.001	0.029
Quercetin-rutinoside hexoside	0.088	-0.036
Quercetin	0.067	-0.085
Quercetin dihexoside	0.030	-0.018
Quercetin malonyl glucoside	-0.096	0.021
Quercetin rhamnosyl hexoside	0.013	-0.044
Kaempferol hexoside	-0.052	0.286
Kaempferol-3-rutinoside	0.062	0.005
Laricitrin hexoside	-0.003	0.073

Catechin	0.064	0.027
Epicatechin	0.104	0.133
Procyanidin dimer 1	0.117	0.099
Procyanidin dimer 2	0.020	0.034
Naringenin hexoside 1	0.115	0.005
Naringenin hexoside 2	-0.125	0.351
Naringenin hexoside 3	-0.171	0.214
Isorharmnetin hexoside	0.113	-0.019
Cyanidin-3-glucoside	0.153	0.085
Cyanidin-3-rutinoside	0.124	0.100
Pelargonidin-3-glucoside	0.161	0.062
Total phenolics	0.503	0.188
**Eigenvalue**	**17.94**	**3.22**
**% variance**	**74.28**	**13.35**
